# Enabling Trade in Gene-Edited Produce in Asia and Australasia: The Developing Regulatory Landscape and Future Perspectives

**DOI:** 10.3390/plants11192538

**Published:** 2022-09-27

**Authors:** Michael G. K. Jones, John Fosu-Nyarko, Sadia Iqbal, Muhammad Adeel, Rhodora Romero-Aldemita, Mahaletchumy Arujanan, Mieko Kasai, Xun Wei, Bambang Prasetya, Satya Nugroho, Osman Mewett, Shahid Mansoor, Muhammad J. A. Awan, Reynante L. Ordonio, S. R. Rao, Abhijit Poddar, Penny Hundleby, Nipon Iamsupasit, Kay Khoo

**Affiliations:** 1 Crop Biotechnology Research Group, Centre for Crop and Food Innovation, Food Futures Institute, Murdoch University, Perth, WA 6150, Australia; 2 ISAAA—BioTrust Global Knowledge Center on Biotechnology, International Service for the Acquisition of Agri-Biotech Applications (ISAAA), IRRI, Los Banos 4031, Philippines; 3 Malaysian Biotechnology Information Centre, Monash University Malaysia, Jalan Lagoon Selatan, Bandar Sunway 47500, Malaysia; 4 Japan Plant Factory Association, 6-2-1 Kashiwanoha Kashiwa, Chiba 277-0012, Japan; 5 Zhongzhi International Institute of Agricultural Biosciences, Research Center of Biology and Agriculture, Shunde Graduate School, University of Science and Technology Beijing, Beijing 100024, China; 6 National Biosafety Committee of Genetically Engineered Products (KKH-PRG), Research Center for Testing Technology and Standards, National Research and Innovation Agency (BRIN), Central Jakarta 10340, Indonesia; 7 Research Center for Genetic Engineering, Research Organization for Life Sciences and Environment, National Research and Innovation Agency (BRIN), Central Jakarta 10340, Indonesia; 8 Australian Seed Federation, 20 Napier Cl, Deakin, Canberra, ACT 2600, Australia; 9 National Institute for Biotechnology and Genetic Engineering (NIBGE), Faisalabad 44000, Pakistan; 10 Crop Biotech Center, Philippine Rice Research Institute, Munoz 3119, Philippines; 11 Sri Balaji Vidyapeeth University, Pondicherry 607402, India; 12 MGM Advanced Research Institute, Pondicherry 607402, India; 13 John Innes Centre, Norwich, Research Park, Norwich NR4 7UH, UK; 14 Biotechnology Alliance Association, Bangkok 10900, Thailand; 15 Regulatory Affairs Manager, Seeds Asia-Pacific, BASF Australia Ltd., 12/28 Freshwater Pl, Southbank, VIC 3006, Australia

**Keywords:** gene editing, genome editing, Cas9, GEd, biosafety, Asia, Australasia, Asia-Pacific, regulations, crops, path-to-market, trade, harmonization, science diplomacy

## Abstract

Genome- or gene-editing (abbreviated here as ‘GEd’) presents great opportunities for crop improvement. This is especially so for the countries in the Asia-Pacific region, which is home to more than half of the world’s growing population. A brief description of the science of gene-editing is provided with examples of GEd products. For the benefits of GEd technologies to be realized, international policy and regulatory environments must be clarified, otherwise non-tariff trade barriers will result. The status of regulations that relate to GEd crop products in Asian countries and Australasia are described, together with relevant definitions and responsible regulatory bodies. The regulatory landscape is changing rapidly: in some countries, the regulations are clear, in others they are developing, and some countries have yet to develop appropriate policies. There is clearly a need for the harmonization or alignment of GEd regulations in the region: this will promote the path-to-market and enable the benefits of GEd technologies to reach the end-users.

## 1. Introduction

Long before the development of Mendelian genetics, farmers selected superior plants from natural populations. Since the early 1900s, the principles of Mendelian genetics have been applied increasingly to improve crop yields, eventually resulting in the ‘green revolution’. More recently, applied crop researchers and plant breeders have sought to improve crops by developing better combinations of genetic material by making use of the increasingly available genomic information, bioinformatics, and molecular biology to identify genes that underlie useful traits and using marker-assisted selection to follow them to develop better combinations of genes for improved traits. 

In parallel with conventional breeding approaches, gene transfer methods have enabled researchers to transfer genes between related or unrelated species to deliver genetically modified (GM) plants or crops, sometimes referred to genetically modified organisms (GMOs) or living modified organisms (LMOs). GM crops now provide more than 10% of the world’s food, but unlike conventional or mutation breeding, both of which are forms of genetic manipulation, the commercial growth of GM crops has been highly regulated, and this has increased the costs of their development. The lack of an international harmonization of regulations for GM crops has been a major factor in holding back their wider implementation [1].

Over the last ten years, so-called ‘New Breeding Technologies’ (NBTs) (also called New Genetic Technologies—NGTs) have emerged, based on the development of targeted mutations using a combination of a double-stranded (ds) nucleases such as Cas9, directed to DNA sequence sites to be cut by guide RNA, making use of the Clustered Regularly Interspaced Short Palindromic Repeats (CRISPR) system employed by bacteria in virus-–defense interactions (e.g., CRISPR/Cas9). Amongst the crop science researchers, there is now an air of excitement about the potential applications of genome-, or preferably, gene-editing (GEd), for crop improvement. In some cases, GEd technology is also described as ‘Precision Breeding’ or ‘Plant Breeding Innovations’. There is now a whole new toolbox of promising applications of GEd with great potential for plant breeders to use and therefore achieve many of their desired outcomes more rapidly. GEd methods are predicted to be game-changing technologies that are poised to revolutionize both basic research and plant breeding [2]. However, it is relevant to note that GEd-based technologies cannot be used to achieve all of the results that can be achieved using GM technologies, because in GEd, current regulations may limit the accessible gene pool. Nevertheless, there are some clear advantages of GEd such as developing non-GM improvements for yield, quality, and abiotic/biotic stress tolerance [3,4]. GEd technology is of particular interest in the developing world where food and nutritional security in a climate change scenario is a real challenge, and GEd can be used efficiently to develop desirable traits in important staple foods [5].

As is often the case, the speed of new scientific developments such as GEd have outpaced policy and regulatory aspects, and this is challenging regulators worldwide. It has become evident that the beneficial applications of NBTs will not be achieved without sensible science-based policies and regulations. In particular, without international harmonization of policies on gene-edited products, the benefits they can confer to society will not be achieved. Scientists may not be in a position to make policies and regulations, but they can influence the process through engagement with policy-makers, politicians, and the public to promote international harmonization of GEd policies and regulations. Only by ensuring internationally compatible, science-based policies for gene-edited crops can the world benefit fully from these exciting new technologies.

The issue of policies that relate to NBTs, and how they could become non-tariff trade barriers, has been recognized with relevant publications that have focused on policies in North and South America, and Europe, but not on the Asia-Pacific region (used here as excluding the Russian Federation, Central Asian republics, Bhutan, Nepal, and the Middle East) including Australasia. As an example, Australia is a major food exporter to Asia, with the top eight grain-importing countries being in Asia, and 70% of exported horticultural produce going to Asia. Without an understanding of international policies on GEd and their harmonization, the trade in products developed using GEd technologies will be severely curtailed.

Here, we provide an overview of the science that underlies GEd and discuss the current regulatory status for gen-edited products, focusing on countries in Asia and Australasia. We discuss the path-to-commercialization and the need to promote the harmonization of national policies, which is vital to enable the future trade of GE products in the region.

### 1.1. Gene-Editing Technology 

Much has been written about the science that underlies GEd, the pace of publications about gene-edited crops is accelerating, and the processes involved have been well-described. In this section, we outline some of the technologies used or available in the GEd toolbox, present some examples of their applications, and provide references to more detailed reviews on this subject.

Although GEd evolved through a variety of approaches such as oligonuceotide directed mutagenesis (ODM), editing using zinc finger nucleases (ZFNs) and transcription activator-like effector nucleases (TALENs), the most widely used GE technology is based on the CRISPR/Cas9 system, which was developed from a bacterial immunity mechanism [6]. The CRISPR-associated endonuclease (Cas9, a dsDNAse) enables what is essentially targeted mutagenesis: the site of the targeted cleavage of DNA is determined by the associated short 19–21 nucleotide single guide RNA (sgRNA). CRISPR/Cas9 facilitates the targeted modification of genetic information at a specific genomic location, enabling the alteration, deletion, or addition of DNA bases at a specific site, which results from DNA repair mechanisms. The cut ends of the DNA sequence may be repaired, most commonly with the loss or addition of a small number of bases (indels) by non-homologous end joining (NHEJ). With the discovery of novel tools of CRISPR and variants of bacterial dsDNA cleaving enzymes, there are recent exciting developments where the PAM sequence requirement for the efficient cleavage of DNA is minimized [7]. Alternatively, with the addition of a donor nucleotide template, nucleotides can be added at the site of DNA cleavage by a process of homologous recombination (HR). The advantage of the CRISPR/Cas9 and associated GEd systems is their relative simplicity and adaptability. A comparison of tools used for GEd (ZFNs, TALENS and CRISPR/Cas9) is provided in [6]. 

Additional techniques in the GE toolbox include base-editing to convert one base into another, modifying the expression levels of target genes by editing promoter sequences, editing genes for transcription factors for multiplexed outcomes, epigenetic changes such as the patterns of DNA methylation or acetylation, and simultaneous targeting of multiple genes to modify multiple or polygenic traits using several sgRNAs at once [8]. The latter enables all the homologous alleles in a polyploid plant, or in a multi-gene family, to be targeted at the same time. Alternative DNAses and gRNAs are also available, some with increased specificity [8].

The application of GEd technology may involve the introduction of a plasmid encoding a dsDNAse such as Cas9 and sgRNA in a T-DNA into the cells of explants or protoplasts (enabling the cells to make their own Cas9 and gRNAs) together with a selectable gene for more efficient selection of potentially edited lines—in this case, selfing of the T0 plants and the selection of segregants null for the editing cassette are required for commercial development. Alternatively, there are methods that do not involve the introduction of T-DNA into the target plant cells, in particular, the introduction of the Cas9 protein and gRNA as a ribonucleoprotein (RNP) complex by particle bombardment, followed by plant regeneration and screening for edited plants. Cas9 is a large protein to deliver into a cell, and its expression via T-DNA is generally more stable and increases the number of edited cells. The use of carbon nanotubes with agrobacterium only for the translocation of GEd components without transformation provides alternatives to the longer process of transgene segregation, particularly in vegetatively propagated crops [9,10,11]. 

It takes more time to identify edited plants after RNP delivery, but this is the only approach that can be used to edit heterozygous clonally propagated species such as potato without losing the original variety. In this case, detecting the presence of targeted mutations in regenerated plants can be achieved by polymerase chain reaction (PCR) and restriction enzyme analysis, targeted Sanger sequencing or genome sequencing. 

GEd technology can also be used in gene drive technology (GDT) where there is a biased inheritance of a gene or genetic element from parent to offspring, resulting in an increase in the frequency of the element until most members of a population contain that genetic element. The potential for GDT in agriculture, driven by mutations created using GE technology, is likely to be developed to eliminate pests, diseases, or weeds from a population, but its discussion was outside the scope of this review.

In summary, GEd technologies can be described as a collection of advanced molecular biology techniques that facilitate precise, efficient, and targeted modifications at genomic loci [12]. 

### 1.2. Summary of Research on GEd Products

**Figure 1:**
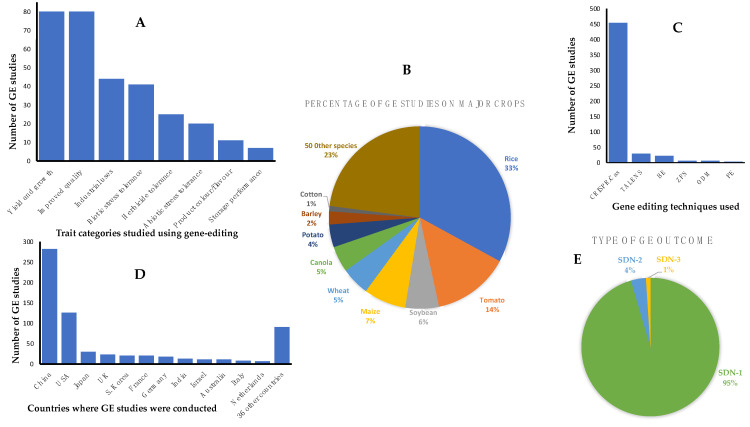
A summary of the published work on GEd to improve traits of plant/crop species. (**A**) The number of GEd studies targeting different traits of crop plants. (**B**) The percentage of GE studies on different species of plants. (**C**) The frequency of use of five GEd techniques in current plant research. BE and PE indicate base-editing and prime-editing. (**D**) Countries where GEd of plants has been undertaken. (**E**) The outcome of GEd type in plant research. (Source: EU-SAGE database; https://www.eu-sage.eu (accessed on 2 July 2022).

It was beyond the scope of this paper to provide a complete list of all published work on GEd for all plants including staple crops such as grains and important horticultural crops. However, the ‘European Sustainable Agriculture Through Genome Editing’ organization (EU-SAGE) has established a database of the latest evidence on GEd applications in plants and many crops. This is a searchable database that describes the plant species, traits, the GEd techniques employed, countries where the work was undertaken and applications covering studies of any crop developed for market-oriented agricultural production as a result of GEd. The database is regularly updated and can be accessed at genome search|EU-SAGE. The traits described are related to plant yield and growth, improved food/feed quality, industrial uses, abiotic and biotic stress tolerances, herbicide tolerance, color, flavor, and storage performance. A summary of the current data on GEd crops in the EU-SAGE database is provided in Figure 1.

From the summary provided in Figure 1, we highlight a few examples of the applications of GEd technology to crop improvement, indicating that GEd, in one form or another, has been applied to all major crop plants including the cereals maize, rice, wheat, barley, sorghum, the staple potato, and industrial crops such as canola and cotton. Other major food crops with data in the EU-SAGE database include soybean, brassicas, tomato, oranges, grapefruit, cassava, flax, cucumber, watermelon and mushrooms, sugarcane, and sugarbeet [6,12,13]. The breeding targets include biotic factors such as resistance to diseases and pests (e.g., resistance to powdery mildew, rice blast, bacterial blight, citrus canker, viruses); quality traits such as the amylose:amylopectin ratio to reduce the Glycemic Index, high oleic acid content, flavor, reduced browning, reduced anti-nutritional factors, improved nutrition such as vitamins A, C, and D; herbicide tolerance; hybrid/breeding systems and maturity dates; grain size, grain number, number of tillers, protein quality, reduced pre-harvest sprouting, reduced allergenicity; improved stress tolerance (e.g., to drought, heat and cold stress), and trait stacking.

### 1.3. Definitions of Gene-Editing—Site Directed Nucleases (SDN)

Gene-editing, in which there is a spontaneous repair at a ds-break (dsB) site whose repair does not introduce external DNA, is usually referred to as Site-Directed Nuclease 1 (SDN-1). If a repair oligonucleotide is incorporated at the dsB, this is referred to as Site-Directed Nuclease 2 (SDN-2), and if a completely new gene cassette is inserted at the dsB, then it is Site-Directed Nuclease-3 (SDN-3). There is general agreement on the definitions of SDN-1 and SDN-3, but SDN-2 can be interpreted in several ways. To date, an SDN-3 product is regarded as a GMO in almost all jurisdictions.

In its broadest sense, SDN-2 is applied to either the insertion of one or a few bases from an HDR oligonucleotide, or the insertion of a complete allele from within the plant’s gene pool, which could have been introduced by conventional breeding. The latter includes allele swopping, which is a standard aim in conventional breeding. The broader definition is much more useful because the question arises on how many nucleotides can be added before an SDN-2 event becomes defined as SDN-3? This aspect is discussed in more detail in Section 3.3.1.

The other question relates to whether recombinant DNA, usually in the form of a Cas9/gRNA cassette with a selectable marker gene, is used initially to improve the efficiency of identifying edited plants amongst the regenerants, followed by selfing and identification of the edited progeny, which do not contain the editing cassette (i.e., do not contain any externally derived DNA).

In considering the current GEd policies of the countries in Asia and Australasia, these issues become apparent, and the current status of GEd regulations in these countries is discussed below.

## 2. The Policies and Regulation of Gene-Editing in Asia and Australasia

Here, we discuss the policies and regulations that relate to the products of GEd technology on a country basis. The aim is to provide an actual or potential pathway-to-market of GEd products where possible in each country: at present, data are not available for Myanmar, Laos, Cambodia, or Vietnam.

### 2.1. GEd Regulations in Australia

The regulation of gene technology in Australia is governed by the Commonwealth Government *Gene Technology Act 2000*, with corresponding State and Territory laws and an Intergovernmental Gene Technology Agreement (https:/www.genetechnology.gov.au, accessed on 2 July 2022). The Act establishes the statutory office holder, the Gene Technology Regulator, to administer the Act and corresponding State and Territory legislation. State and Territory governments take part in governing the scheme through the Gene Technology Minister’s Meeting and Gene Technology Standing Committee, which support a nationally consistent regulatory system for gene technology. How the Act is implemented is described in the *Gene Technology Regulations 2001* including the duties of the Office of the Gene Technology Regulator (OGTR). 

**Figure 2:**
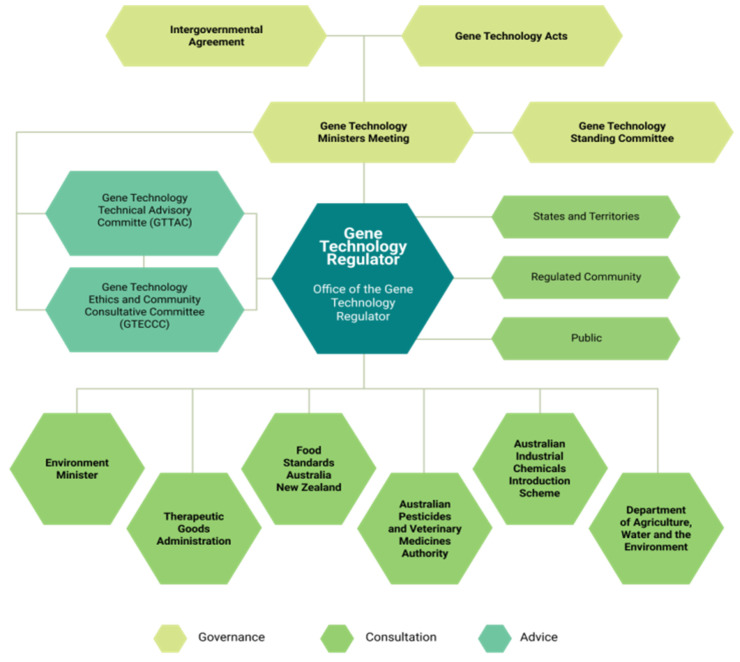
An overview of the bodies that administer or provide information or advice to the Gene Technology Regulator in Australia (https://www.ogtr.gov.au/; accessed on 2 July 2022).

An overview of the scheme is provided in Figure 2, which summarizes how the scheme is governed, the groups that provide advice, and who takes part in the consultations.

To undertake work with GMOs, the Gene Technology Regulator and staff at the OGTR administer the Act and regulate the use of GM organisms. The Office is based in the Commonwealth Government Department of Health, with the remit to ensure that dealings are safe and well-managed to protect human health and the environment. It covers all aspects of gene regulation including health, the environment, industrial applications, and agriculture. 

The duties of the Regulator include administering licenses, developing policy principles and guidelines, codes of practice, ensuring compliance with the legislation and providing advice on gene technology to Government ministers, other agencies, and the public (https://www.ogtr.gov.au/; accessed on 2 July 2022).

The *Gene Technology Act 2000* is restricted to governing living organisms: the standards for the safety, content, and labelling of food is the responsibility of Food Standards Australia New Zealand (FSANZ). Although the scheme has withstood the test of time, the OGTR recognized that new technologies were outpacing the regulations, and to ensure the legislation was fit-for-purpose, a series of reviews of the scheme were undertaken, with the aim of future-proofing it for current and future scientific developments. 

The Gene Technology Regulations can designate organisms that are not GMOs (Schedule 1). Natural mutations and mutations induced by chemical or irradiation treatments are classified as ‘not GMOs’, but the status of SDN-1 (targeted changes with unguided repair), template guided repair with oligonucleotides (SDN-2), or with longer templates (SDN-3) was not clear. Following the review, the outcome was that only SDN-1 products were excluded from the regulations, but organisms edited by SDN-2 or ODM and SDN-3 were regulated as GMOs. If no external DNA is used to generate the edited organism (e.g., RNP process or transient expression of the editing cassette), the organisms are not GMOs. Similarly, if an integrated editing expression cassette is no longer present in null segregants, then the resultant organism is not a GMO. Table 1 summarizes the current status of the regulation of GEd organisms in Australia.

What this means is that organisms developed from SDN-2 and SDN-3 treatments are still regulated as GMOs in Australia. This result puts Australia at odds with some other developed countries in which SDN-2 edits are not classified as GMOs if they introduce sequences from plants in the same gene pool, and which could have been developed by conventional breeding techniques. In effect, the valuable opportunity for allele swopping in breeding cannot be conducted without a product being designated as a GMO.

**Figure 3:**
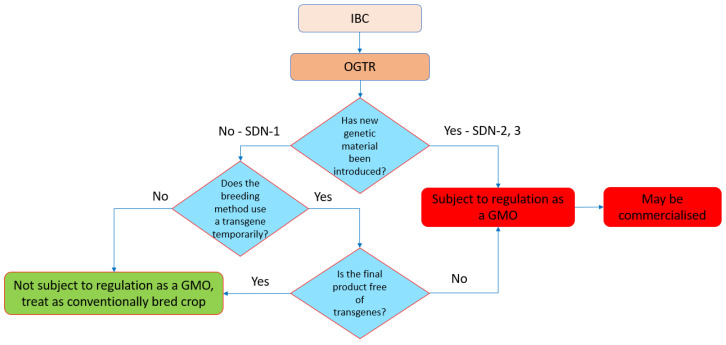
A summary of the pathways to the deregulation of SDN-1 GEd products in Australia.

The overall scheme describing the pathway for the deregulation of GEd products in Australia is shown in Figure 3. 

#### 2.1.1. Modernizing and Future-Proofing Gene Technology Regulatory Schemes in Australia

As previously described, the importation and cultivation of GMOs in Australia is regulated through a nationally consistent legal scheme, the *Gene Technology Act 2000* and the *Gene Technology Regulations 2001*. The Act is administered by the Gene Technology Regulator, who is responsible for making decisions on whether to approve field trials and the commercial release of GM crops. GM products are regulated by a number of authorities with specific areas of responsibility (Figure 3) such as FSANZ, which sets the standards for the safety, content, and labelling of food.

Following a Technical Review of the Gene Technology Regulations, the regulatory status of some GEd techniques in Australia was clarified (Table 1) (i.e., organisms modified using site-directed nucleases without templates to guide genome repair (i.e., SDN-1) are not regulated as GMOs). These organisms are treated the same as those resulting from conventional breeding process, and no consultation with the Regulator is required. If a template is used to guide genome repair (i.e., SDN-2 and SDN-3), the resulting organisms are GMOs, as are organisms modified using ODM. However, there remains a lack of regulatory clarity when it comes to foods derived from gene technology. For example, a product developed using SDN-1 is not a GMO for cultivation purposes; however, whether it is regulated as a GM food is subject to the outcomes of the ongoing FSANZ review of food derived from new breeding techniques.

In December 2020, the Australian Department of Health launched a review paper on ‘Modernizing and Future-Proofing Australia’s Gene Technology Regulatory Scheme’ (https://www.genetechnology.gov.au/resources/publications/2017-review-consultation-regulation-impact-statement-modernising-and-future-proofing-national-gene-technology-scheme; accessed on 2 July 2022). This paper presented three options for consultation:

Option A: Status quo—no changes.
Option B: Risk-tiering model—dealings classified according to their indicative risk.
Option C: Matrix model—the nature of the dealing determines its classification.

**Figure 4:**
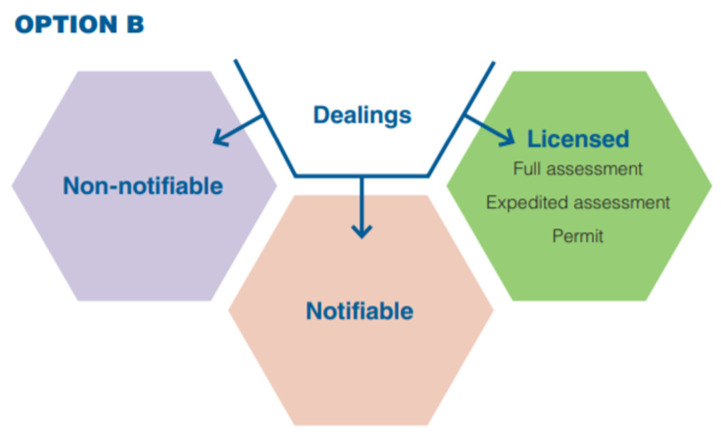
Preferred Australian model for assessing the risk of GEd foods. Option B: Risk-tiering model—where dealings are classified according to their indicative risk (https://www.genetechnology.gov.au/resources/publications/2017-review-consultation-regulation-impact-statement-modernising-and-future-proofing-national-gene-technology-scheme; accessed on 2 July 2022).

Option B, a risk-tiering approach, was the preferred option of most responders and was recommended for adoption in the subsequent Decision Regulatory Impact Statement. Option B enables dealings with GMOs to be distinguished based on indicative risk (i.e., enabling a proportionate risk response). This approach is summarized in Figure 4 below:

For example, the gene technology used to create the GMO would be a relevant consideration. If a specific gene technology (i.e., some types of GEd) presents a very low risk and a case-by-case assessment is not required, then these dealings could be eligible for one of the ‘lighter-touch’ pathways. However, under this option, even those dealings classified as non-notifiable are still considered as a ‘GMO’ and are not ‘excluded’ from regulation. This is significant compared to the SDN-1 exclusion described earlier.

#### 2.1.2. Food Derived from New Breeding Techniques

FSANZ is currently undertaking a review on its regulatory approach to food derived from new breeding techniques (NBT food) (FSANZ 2019, Review of food derived using new breeding techniques, Final Report). The starting point for the review is that the need for the pre-market assessment of food derived from NBT food is essentially a question about risk, and how NBT food compares to conventional food. If it can be demonstrated that NBT food is equivalent in risk to conventional food, then it may be argued that a pre-market safety assessment is unnecessary. FSANZ suggests that when assessing the risk(s) from NBT food, the size of genetic change, whether it was intended or not, and the method used to effect the genetic change are irrelevant considerations (FSANZ 2019).

According to FSANZ (2019), the crucial factor from a food safety perspective when any genetic change is made is the impact of that change on the food. If a genetic change is made using a new breeding technique, and the introduced change has not resulted in new or altered product characteristics compared to conventional food, then FSANZ concludes “the NBT food will carry the same risk as the equivalent conventional food”.

This provides a clear basis for excluding these foods from a requirement for the pre-market safety assessment as a GM food.

### 2.2. GE Regulations in New Zealand

Agricultural exports constitute a large part of New Zealand’s (NZ) economy, and at present, no GM crops are grown. GM organisms are regulated by the *Hazardous Substances and New Organisms (HSNO) Act 1996*. In Australia, this Act defines what is a GMO, where GMO means, unless expressly provided otherwise by regulations, any organism in which any of the genes or other genetic material (a) have been modified by in vitro [not defined] techniques; or (b) are inherited or otherwise derived, through any number of replications, from any genes or other genetic material which has been modified by in vitro techniques, and organisms that are not genetically modified. 

In 2014, the NZ Environmental Protection Authority ruled that plants produced using GEd methods, where no foreign DNA remained in the edited plant, would not be regulated as GMOs. However, following a challenge in the High Court, this decision was overturned so that NZ currently regards all products of GEd as GMOs [14].

### 2.3. The Regulatory Status of GEd Produce in Japan

The Japanese government positions GEd as a key innovative technology. In 2014, the Government started funding research with the goal of bringing products developed through GEd to market, under the ‘Cross-ministerial Strategic Innovation Promotion Program’ (SIP Program) [15]. Discussions on the related policy were then initiated since the Government recognized that a science-based and applicable policy was vital for the commercialization of GEd products. After five years of discussion, which involved inviting many expert opinions and holding public hearings, the Government finally published the handling policy in 2019. Since GM technology may be used during the early stages of GEd techniques, and GEd can result in different types of products, depending on whether or not a repair template is used as well as the nature of the template used, the Government discussed each case separately, and determined what type of products were or were not subject to existing GMO regulations. Table 2 summarizes the GEd handling policy under the *Food Sanitation Law*, the *Feed Safety Law*, and the *Cartagena Law* (the environmental safety law) and each responsible Ministry. There is a discrepancy in the handling policy resulting from different definitions of a GMO under each relevant law. 

**Figure 5:**
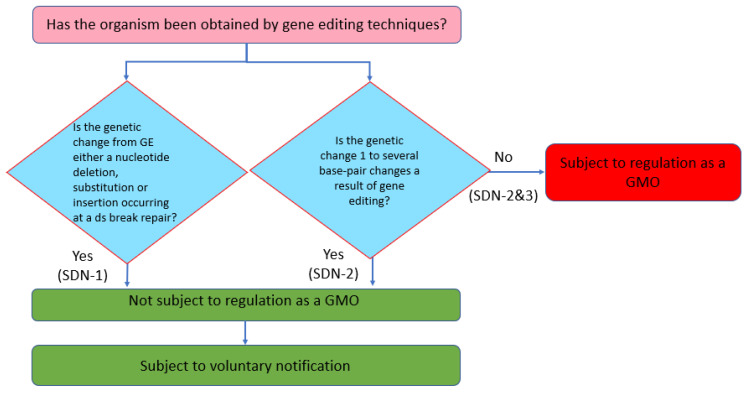
Policy decisions for GEd/GM products in Japan by the *Food Sanitation Law* and *Feed Safety Law*.

The definition of a GMO under the *Food Sanitation Law* and the *Feed Safety Law* can be described as ‘an organism obtained through recombinant DNA techniques’. Although the definition of a GMO is process-based, produce-based risk assessments have also been undertaken for GMOs in Japan. Therefore, in determining the handling policy for GEd products, the Ministry of Health, Labor, and Welfare (MHLW) considers whether or not the GEd product can be distinguished from a product obtained through spontaneous or induced mutation: if they are indistinguishable, the risk of a GEd product should be within that of the product obtained through conventional breeding. Hence, if the genetic change induced by GEd is either a nucleotide deletion, substitution, or insertion resulting from the repair of a double-strand break (i.e., SDN-1), such a product is determined as not being subject to regulation as a GMO (Figure 5). In addition, if the genetic change as a result of GEd is up to a ‘few base pairs’ (i.e., SDN-2), the product is not subject to regulation as a GMO. However, if the genetic change is larger than a few base pairs, the product is regulated as a GMO regardless of the source of the template DNA (i.e., SDN-2 and 3).

**Figure 6:**
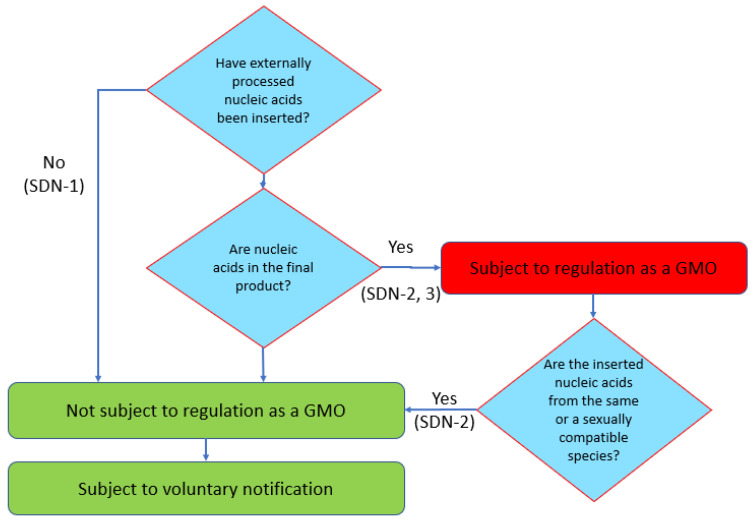
Pathway to the deregulation of GEd products under the Cartegena Law (Environmental Safety) in Japan.

The definition of a GMO under the environmental safety regulation (*Cartagena Law*) is that as a ‘living modified organism is an organism that possesses extracellularly processed nucleic acids or its replicate excluding the case that the nucleic acids are from the same species (self-cloning) or from sexually compatible species (natural occurrence)’. Consequently, if the final product does not possess nucleic acids that have been processed outside the cell, the product is not subject to regulation as a GMO (Figure 6). On the other hand, if a template DNA is used so that the final product contains extracellularly processed nucleic acids, the product is subject to regulation as a GMO. However, since the *Cartagena Law* does not regulate a product, if the extracellularly processed nucleic acids are from the same species or from sexually compatible species, the same rule applies as for a GEd product. This means that GMO/non-GMO determination of SDN-2 and 3 type products is made on a case-by-case basis. 

Because GEd is a new technology and is evolving, the Japanese Government still requests developers to submit ‘voluntary’ pre-marketing notification documents, even if the product is not subject to GM regulation. This notification allows the Government to further confirm that products derived from GEd are as safe as products obtained through conventional breeding, and to gather information on what products are on the market so that the Government can take additional action if needed. The request for voluntary notification also reflects the consumers’ concerns that GEd products would be commercialized without any checking or tracking system.

The information that should be included in notification documents to MHLW are as follows:

- Crop, variety, use of the product, and purpose of use.
- The GEd method used, and details of the modification.
- Absence of foreign DNA (using appropriate methods, including Southern blot, Next Generation Sequencing, and PCR.).
- Confirmation that the change (including off-target changes) in DNA does not produce new allergens or increase known toxins (i.e., MHLW currently expects a developer to conduct (i) analysis of off-target edits, and (ii) homology searches to known allergens and toxins not only at the targeted site, but also an off-target edit site, if identified).
- Any change of metabolites relating to a targeted metabolic pathway.
- Year and month of launch (after commercialization)

The information required by the MOE and MAFF is basically the same, except that discussion of any possible influence on biological diversity is required instead of a discussion on allergenicity and toxicity. The notification document is posted on each relevant Ministry’s website immediately after submission. By May 2022, three products had undergone the voluntary pre-marketing notification process in Japan: these were high-GABA tomato, sea bream with more meat and better feed efficiency, and tiger pufferfish with faster growth and better feed efficiency. Several public research projects are in progress, with more products expected to be notified soon.

### 2.4. The Regulatory Status of GEd Produce in China

Chinese researchers have a strong track record of research on GEd crops, as evidenced by the fact that China holds more patents on plants than any other country [16], but to date, no GEd crop products have been commercialized [17]. The safety evaluation of GEd crops has not been subject to the same regulations as GMOs since January 2022, when the Guidelines for Safety Evaluation of Agricultural Gene Editing Plants (Trial Edition) was issued by the Ministry of Agriculture and Rural Affairs (MARA, formerly MOA). However, in the four following regulatory steps, which include crop variety registration, seed production evaluation, seed business evaluation and processing evaluation, GEd crops are still regulated as GMOs.

#### 2.4.1. Current GEd Regulatory Status in China

There are five steps for a GEd product to proceed from the laboratory to the market. These are: safety evaluation, crop variety registration, seed production evaluation, seed business evaluation, and processing evaluation. Except for the first step, Chinese oversight of GEd plants is based mainly on the *Regulations on the Safety Management of Agricultural Genetically Modified Organisms* (referred to as the ‘*Regulations*’) issued by the State Council in 2001. Article 3 states ‘that an agricultural GMO denoted in these Regulations relates to animals, plants, micro-organisms, and their products in which genetic engineering technology was used to change the genome composition for agricultural production or agricultural product processing’. This means that all crops and their products obtained through GEd technology are classified as agricultural GMOs, and are included in the safety management of agricultural GMOs according to law.

**Figure 7:**
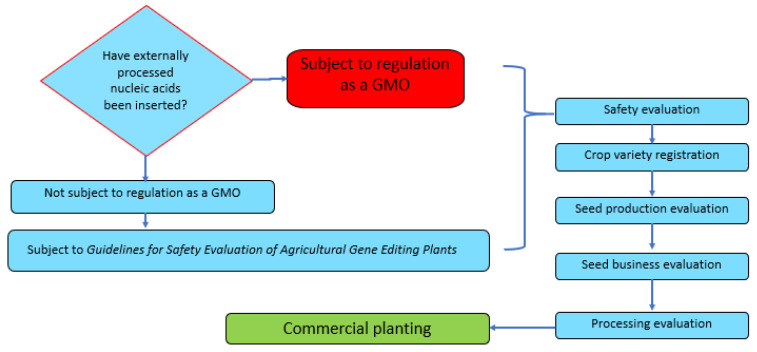
The path to commercialization for GM and GEd crop plants in China.

There are three specific administrative measures and related guidelines that support the *Regulations*. In 2002, the then Ministry of Agriculture (MOA) issued the *Administrative Measures for the Safety Evaluation of Agricultural Genetically Modified Organisms*, which have since been revised three times, in 2004, 2016, and 2017. The *Administrative Measures for the Import Safety of Agricultural Genetically Modified Organisms* have been revised, twice in 2004 and 2017. For the *Administrative Measures for the labelling (‘marks’) of Agricultural Genetically Modified Organisms*, a revision was made in 2004. These revised versions made more specific provisions on the safety evaluation, import safety approval, and identity management system for the safe management of agricultural GMOs. In January 2022, MARA formulated and published new *Guidelines for Safety Evaluation of Agricultural Gene Editing Plants* (Trial Edition). It further standardized the safety evaluation management of agricultural GEd plants. This is a milestone for R&D using NBTs and industrial promotion in China. As a result, the current path-to-commercial growth of crop plants described in Figure 7 is expected to pave the way for more crops with advanced traits to be commercialized. 

#### 2.4.2. China’s Government Organizations Responsible for GEd Monitoring

The main supervising authorities of GEd/GM procedures in China are MARA, together with its subordinate agricultural departments, which are specifically responsible for the supervision and management of the safety of agricultural GEd products and GMOs. MARA is also responsible for reviewing and issuing Safety Certificates, Variety Certificates, Seed Production Permits, Seed Business Permits, and Processing Permits for agricultural GMOs. In addition, other departments including the Science and Technology Departments, Development and Reform Commission, Ministry of Commerce, General Administration of Customs, and Market Supervision Departments of China, are also responsible for the R&D investment, market access, domestic circulation, import and export, and labelling management. For other organisms (such as trees), authorities such as Ministry of Forestry have the power of oversight for relevant GM products in their areas.

Although the Ministry of Ecology and Environment (MEE) appears not to be concerned with the domestic safety regulation of GEd products, it is responsible for China’s national responses to the secretariat of the Convention on Biodiversity Diversity (CBD), and is active in environmental risk assessment and management in China. 

#### 2.4.3. Progress toward Deregulation of GEd Products in China

In China, there have been rapid developments in GEd technology applied to crops, especially based on the CRISPR/Cas9 system [12,18]. There is currently an ongoing discussion on the regulation of GEd produce, which has focused mainly on the supervision of their safe use to support the current increasing trend of applications for GEd technologies for crop improvement [19]. Based on the rapid developments of GEd technologies, there is an urgent need to improve public trust and establish a science-based regulatory system for GEd R&D and products. The current desire by the Chinese scientific community is for regulatory bodies to implement a consultation system that will assess GEd products on a case-by-case basis, especially for products of SDN-1 and SDN-2 technology, bringing them closer in line with international standards [17]. 

### 2.5. The Regulatory Landscape for GEd in India

In 1989, well before there was global consensus on regulating biotechnology through the *Cartagena Protocol*, India enacted its domestic biotechnology regulatory framework through the ‘*Rules for the Manufacture, Use, Import, Export, and Storage of Hazardous micro-organisms/Genetically engineered organisms or cells’* (*Rules* 1989) under the *Environment (Protection) Act of* 1986. The *Rules* regulate activities involving genetic engineering from the bench to market through six competent agencies at the *process*, *product*, and *processed* ‘*product thereof’* levels. ‘*Product thereof’* includes any item that either contains GMOs or are derived from a GMO, but may not contain GMO in the final product (e.g., oil derived from a GMO is known as ‘product thereof’). These agencies are either regulatory or advisory and operate at different levels, starting from institutions to the central government agencies. Research activities including the import, export, and transfer of materials and contained experiments are monitored by *Institutional Biosafety Committees* (IBSC) at the institutional level and regulated by the *Review Committee on Genetic Manipulations* (RCGM) at the federal level, and operate from the Department of Biotechnology (DBT). In contrast, the *Genetic Engineering Appraisal Committee* (GEAC), the apex regulatory body, operates from the Ministry of Environment, Forest and Climate Change (MoEF&CC), and approves confined field trials, the environmental release of GM products, and the large-scale commercial import and export of GM materials. 

With the introduction of various forms of GEd, in which processes such as SDN-1 and SDN-2 involve deletion or minor edits in the genome, similar to products that have mutations identical to those found in nature, regulators have debated whether these changes should be regulated, and if so, how. In India, there has been a clear picture where *Rule* 3 of the *Rules* 1989 defined genetic engineering more broadly to cover the deletion or insertion of a nucleotide or a DNA sequence coding for a gene or regulatory element, which may result in the development of a trait that may or may not exist in nature. As a result, emerging technologies of GEd were essentially regulated under *Rules* 1989 at the process and product levels.

However, following inter-ministerial and stakeholder consultations, and considering the available options, government actions were consolidated under *Rules* 1989, which led to a more sensible regulatory approach to GEd technologies. Several guidelines and office memoranda were then issued under the *Rules* 1989, which required Indian regulators to adopt a process-weighted regulatory mechanism where regulatory decentralization and deregulation were introduced, based on the process of modification and the product, and depending on the presence of exogenous DNA. As a result, laboratory experiments involving SDN-1 modifications now require no approval, except that the IBSC needs to be informed. In contrast, SDN-2 and SDN-3 experiments require the approval of the IBSC and RCGM, respectively. 

Recently, the MoEF and CC have deregulated all SDN-1 and SDN-2 forms of GEd products if they are free from exogenous DNA. However, experimental plant work on GEd is still regulated by the IBSC and must be reported to the RCGM, and be undertaken under containment conditions until the plant is free from exogenous DNA. To obtain the deregulated status, an applicant needs to submit information to the IBSC for appraisal and inform the RCGM. Significantly, all SDN-1 and SDN-2 plants are exempt from further biosafety trials under field conditions as well as from food and feed safety assessments, and require no approval from GEAC for commercialization. In contrast, editing technologies with a footprint of exogenous DNA (i.e., SDN-3 is still regulated at the process and product levels as for ‘traditional’ GMOs).

**Figure 8:**
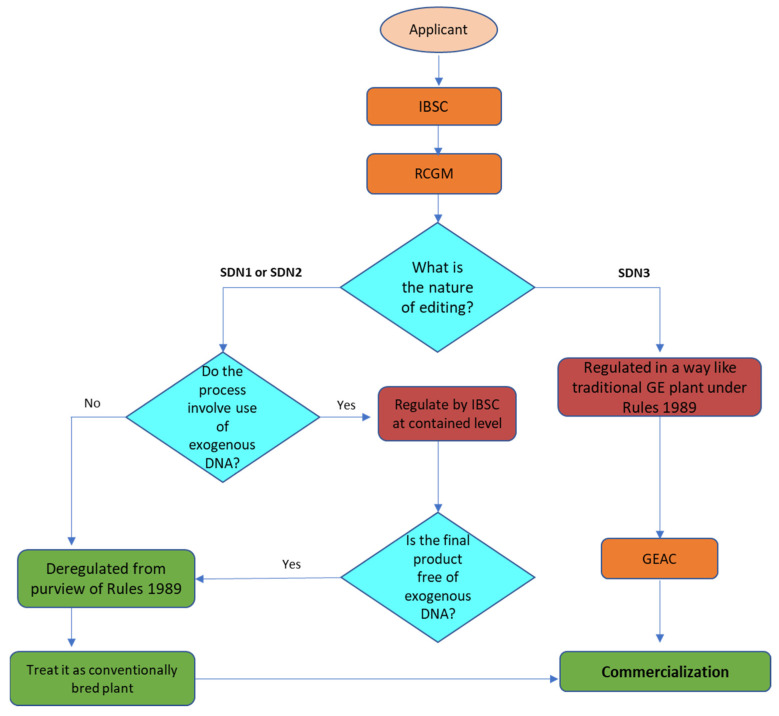
The regulatory pathway for gene-edited plants in India.

To facilitate assessment of the type of SDN modification in a GEd product, the DBT has guidelines for the type of required Risk Assessment and Risk Management. The assessment is conducted by the IBSC and RCGM based on information submitted on the plant biology, programmable nuclease, template nucleotide sequence(s), molecular characterization, editing and selection methods, possible off-target mutations, and stability of edits over generations. Although the guidelines give a much clearer roadmap involving experimentation with GEd plants, they do not specify the number of base changes allowed for SDN-2 GEd, that is, editing having detectable edit footprints and the source of the template DNA. Furthermore, in parallel with the deregulation of SDN-2 and its definitions in the guidelines, altered expression profiles due to defined footprint edits are considered as an allelic form comparable to those available in a primary/secondary gene pool. It is possible that the latter has made biosafety assessments quite questionable, especially when the source of the template DNA crosses species barriers. Thus it has not specified the regulatory status for plants with inserted detectable nucleotides obtained through SDN-2 methods, or imported GEd seeds for experimentation, cultivation, or food/feed use. The regulatory pathway for GEd in India is shown in Figure 8.

Despite some aspects that still need clarity, good progress has been made, especially because India has not yet assessed or provided any commercial approval for GEd plants. However, it is expected that the current roadmap will facilitate R&D being undertaken on GEd in India (Table 3) and those planning to use the technology to improve crop plants. 

### 2.6. The Regulatory Status of GEd Produce in Pakistan

Pakistan has a population of over 220 million. With one of the highest population growth rates, the country will require technologies such as GEd to enhance the yield, quality, and nutritional value of food and fiber crops, which are the mainstay of its economy. To date, different institutions in Pakistan are working in collaboration with researchers in other countries to improve important crops such as wheat, rice, cotton, potato, oilseed brassicas, soybean, and tomato using various GEd tools (Table 4). 

Being a signatory of the *Convention of Biological Diversity* (CBD) and *Cartagena Protocols*, Pakistan is obliged to address the potential risks or hazards posed by genetically modified organisms (GMOs) before releasing them into the environment. For this purpose, the *Ministry of Environment (now named Ministry of Climate Change*) constituted a *National Biosafety Committee* (NBC) to consider issues regarding GMOs and their products before conducting controlled laboratory research, field studies, and commercial release. To date, gene-edited organisms (if a foreign gene is present) are treated as GMOs in accordance with the guidelines provided in the *Pakistan Biosafety Rules*, 2005 issued by the *Ministry of Environment* under the umbrella of the *Pakistan Environmental Protection Act*, 1997. In contrast, if a foreign gene is absent or segregated out, then the edited plant will not be subject to GMO regulations. A meeting of all stakeholders was organized by the National Institute for Biotechnology and Genetic Engineering (NIBGE) to discuss regulations for the release of GEd products in Pakistan. The consensus was that the GEd policies of Japan and Australia will be adopted. The *Institutional Biosafety Committee* (IBC) considers all GEd cases and submits recommendations on the status of projects or research outcomes as GMO or not to the NBC. This follows very much a policy that SDN-1 and SDN-2 products will not be regulated as GMOs, whereas when a foreign gene is present, the product will be treated as a GMO (SDN-3). 

**Figure 9:**
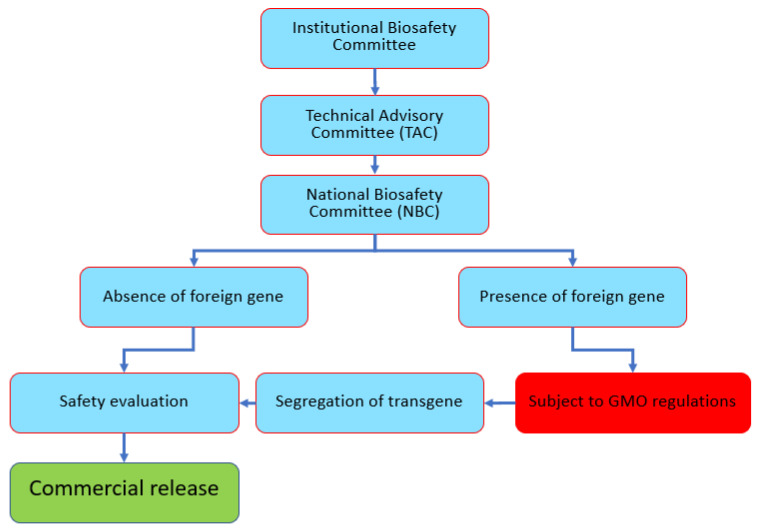
The administrative framework for commercial release of GEd or GM crops in Pakistan.

According to the proposed guidelines, three tiers have been established for monitoring and implementation, namely the IBC, the Technical Advisory Committee (TAC), and the NBC. First, the Principal Investigator (PI) and researchers of a GEd project are responsible for the safety, hazards, and risks to themselves and the community. The IBC undertakes the initial risks assessment and later monitors and inspects the processes involved in the project. After evaluating the application and the available resources, keeping in mind any associated risk or qualification of the responsible PI, the IBC will forward the application to the TAC. The TAC plays a major role in evaluating and reviewing all the applications more technically for licensing. The TAC ensures that the GMOs or any product under consideration go through the necessary risk assessment according to the guidelines provided in the *Pakistan Biosafety Rules*, 2005. Third, the NBC, operating directly under the Ministry of Environment, oversees the overall monitoring, safety, and risk assessment/management of the laboratory, fieldwork, and commercial release of GMOs and their products (Figure 9).

Because of the flexible regulatory procedures for GEd crops in Pakistan, academic and research institutes have reported the use of the CRISPR-Cas systems in crops such as rice, potato, wheat, and cotton (Table 4). In basmati rice, for example, knockout mutants have been developed to counter bacterial blight disease, caused by *Xanthomonas aryzae pv. oryzae* (Xoo) by disturbing effector binding elements (EBEs) in the promoter region of the OsSWEET14 gene [20]. Similarly, a CRISPR-Cas13-mediated multiplexing approach has been used in potatoes to confer resistance to multiple strains of potato virus Y (PVY) [21]. For wheat, the phytic acid content has been decreased to biofortify the crop by enhancing the accumulation of iron and zinc in mature grains [22]. NIBGE is carrying out broad-spectrum R&D work on major crops such as wheat, rice, soybean, and cotton to improve the yield, quality, and develop resistance to biotic/abiotic stresses through CRISPR-Cas systems in commercially grown cultivars. Despite all the work in progress, as no GEd crop has yet progressed through the system or gone to national trials. However, the pace of ongoing initiatives by the Government shows the seriousness of promoting and accepting GEd crops to combat adverse challenges posed by climate change and reduced arable land.

### 2.7. The Regulatory Status of GEd Produce in Thailand

At present, Thailand does not have specific laws to regulate GM crops. To undertake such GM studies, researchers need to follow the *Biosafety Guidelines for Work Related to Modern Biotechnology* (2016). The guidelines embrace all work related to gene manipulation employing recombinant DNA (rDNA) technology for all purposes including the development of transgenic plants, animals, and micro-organisms, production of vaccines, commercial and industrial manufacturing of rDNA-derived products, and the release of transgenic materials and products into the environment.

The importation of GM crops and their products is evaluated under the *Plant Quarantine Act* (1964) (amended in 1994): in 1994, the *Department of Agriculture, Ministry of Agriculture and Cooperatives*, made a “Ministerial Declaration” prohibiting the import and transit of all transgenic plants, unless permission is granted by the Director-General of the *Department of Agriculture*, and only for experimental purposes. For foods derived from GM crops, the Ministerial Order (2002) under the *Food Act* 1965 (Amendment 2002) requires the labelling of GM products.

Additional guidelines were developed later including the Biosafety Guidelines for Contained Used of GM Micro-organisms at Pilot and Industrial Scales, Biosafety Guidelines for Plants Carrying Stacked Genes and Their Derivatives and Guideline on Plant Biosafety in Research Greenhouses, while the precautionary principle was adopted for biosafety actions (https://www.tei.or.th/file/library/2020-Progress-Biodiversity-eng_30.pdf; accessed 15 July 2022).

New regulations are being considered at present. These are:

1. The *Draft Genetically Modified Foods Regulation*, which includes the regulation lists of those GM plants that have passed food safety assessment by the risk evaluation agencies designated by the Thai Food and Drug Administration. Food products consisting of or produced from GM food (GMF) ingredients are prohibited from production/processing, retail or importation into Thailand unless they have undergone food safety evaluation. New GMFs must be assessed by designated risk evaluation agencies.
2. The *Draft Biodiversity Law*, which includes regulating living modified organisms (“LMOs”) that are alive and capable of transferring genetic material to subsequent generations. Under this draft, LMOs are strictly prohibited from being released into the environment unless they are on the Release List, and such a release is handled according to the requirements stipulated in the draft and its secondary legislation. To be announced on the Release List, the applicant must submit an application to the Competent Authority, together with a biosafety risk assessment report (https://www.lexology.com/library/detail.aspx?g=767d7504-faae-42d1-b0e1-a4b7854b296c; accessed 15 July 2022).
3. The *Draft Biosafety Assessment Guidelines for Genome Editing Technology*, which includes three types of GEd technology, and details of the minimum requirements for a food and feed safety assessment (https://www.isaaa.org/webinars/2021/gethai/ppt/Policy%20Considerations%20of%20Genome%20Editing%20in%20Thailand%20-%20Chalinee%20Kongsawat.pdf). The draft *Biodiversity Law*, which covers research, field trials, and the commercialization of GM plants, animals, and micro-organisms, will be sent to the Cabinet for approval by the end of 2022 (https://apps.fas.usda.gov/newgainapi/api/Report/DownloadReportByFileName?fileName=Agricultural%20Biotechnology%20Annual_Bangkok_Thailand_10-20-2020).

#### 2.7.1. Government Organizations Responsible for GM/GEd Regulations in Thailand

There are four main government agencies involved in the regulation of research and products of agricultural biotechnology. These are: 

Department of Agriculture (DOA), Ministry of Agriculture, and Cooperatives (MOAC), responsible for regulating imported GM and GEd seed for planting, conducting GM/GEd research and development, and risk assessment

The National Center for Genetic Engineering and Biotechnology (BIOTEC), Ministry of Higher Education, Science, Research and Innovation (MHESI), is responsible for developing technical guidelines and providing technical advice under the Biosafety Program 

The Ministry of Natural Resources and Environment (MONRE) is responsible for drafting the National Biosafety Law (Biodiversity Law) and is the national focal point for the Convention on Biological Diversity (CBD) and Cartagena Protocol on Biosafety (CPB) and The Thai Food and Drug Administration (TFDA), Ministry of Public Health (MOPH) is responsible for regulating and monitoring the use of GM/GEd food including labelling and regulating the imports of GM/GEd-contained food products. 

#### 2.7.2. Examples of GM/Ed Crop Products Being Commercialized in Thailand

Although no GM or GEd crops have been approved for field trials or commercial cultivation in Thailand, the importation of GM crops does take place, and is limited to corn, soybean, and cotton for feed and industrial use.

GEd technology in Thailand is defined as a type of genetic engineering in which a nucleotide sequence is inserted, deleted, or replaced in the genome of a living organism using engineered nucleases, thus, it is regulated in the same manner as GM crops. GM/GEd products must undergo risk assessment before approval for commercial release. However, for SDN-1, there are only three components that need to be considered: a comparison of the nucleotide sequence difference between a GEd product and its unmodified counterpart, the product specification, and an off-target analysis. According to the *Draft Biodiversity Law*, to take GM/GEd crops to the market, they must be approved after risk assessment by the relevant authority and then included on the Release List. According to the draft GEd regulations, GEd products need to be assessed on a case-by-case basis. Minimum assessment requirements are imposed on SDN-1 products whereas SDN-3 products are assessed rigorously. 

Since the *Draft Biodiversity Law* is still under consideration by the Cabinet, and the secondary laws are under development, the picture is not clear enough to provide a certain path-to-market for GEd crops in Thailand.

### 2.8. The Regulatory Status of GEd Produce in the Philippines

The Philippines was the first ASEAN country (1990) to initiate a biotech regulatory system for GMOs. It recognized the value of modern biotechnology and the need to develop policies that could evolve with technological developments. To develop policies for the expected NBT products (including GEd), imported or from local research, in January 2018, the Department of Agriculture—Biotech Program Office (DA-BPO) initiated a Study Group to review the status of eight different NBTs (SDNs, ODMs, Cisgenesis, Intragenesis, RNA-dependent DNA methylation (RdDM), grafting with GM material, reverse breeding, agro-infiltration and synthetic genomics) to provide science-based policy recommendations on how such products should be treated. The existing GMO regulations (Joint Department Circular no. 1, series of 2021 or JDC1, s2021) were the defining departure point. The Study Group findings and consultation meetings (February–September 2018) generated “A Review of the New Plant Breeding Techniques (NBTs) from the Viewpoint of Regulation”, which was submitted to the DA-BPO (in November 2018) and to the National Committee on Biosafety of the Philippines (NCBP) in May 2019. The NCBP then commissioned an Ad Hoc Technical Working Group (TWG) (from June–December 2019) to resolve the regulation of such products and this resulted in a policy on NBTs, NCBP Resolution No. 1 series of 2020, also known as “*The Regulation of Plant and Plant Products Derived from the Use of Plant Breeding Innovations (PBIs) Or New Plant Breeding Techniques (NBTs)”*, issued in April 2021. 

**Figure 10:**
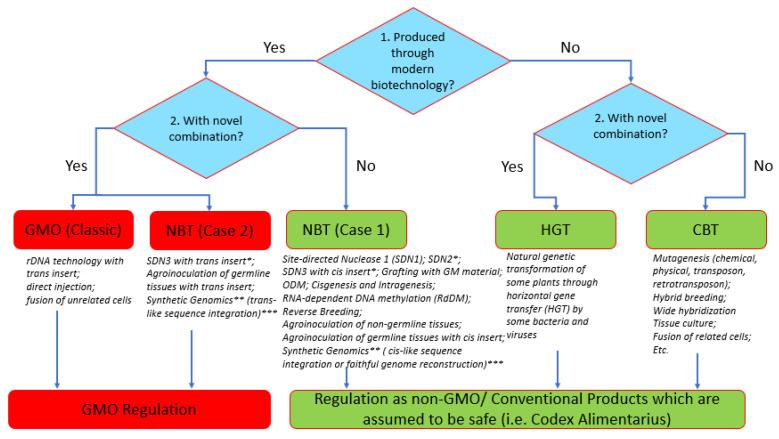
Decision tree for NBT products from the Ad Hoc Technical Working Group of the National Committee on Biosafety of Philippines (NCBP). * includes insertion using the CRISPR-CAS with Prime Editing; ** not to be confused with Synthetic Biology, which specializes on sequences/genetic elements (e.g., unnatural base pairs) in the genome that are not found in nature (beyond novel combination); *** relates to a largely synthetically assembled genome).

The resolution synonymized NBTs with plant breeding innovations (PBIs). In summary, products of NBTs can be (a) GMO, if, as defined under Executive Order (EO) 514 (2006), they contain a novel combination of genetic materials obtained by modern biotechnology. “Novel combination” was defined by the Ad Hoc TWG as a “resultant genetic combination/change in a living organism that is not possible to obtain through conventional breeding”, or (b) non-GMOs or conventional products, if they do not contain a novel combination of genetic material. Only GMOs will be regulated under JDC No. 1, whereas their non-GM counterparts are not regulated under this JDC, but are still subject to regulations normally applied to conventional plant products. To facilitate understanding of the techniques covered by the policy, a decision tree was created (Figure 10). In the tree, it is clear that GEd can lead to the formation of a non-GMO (PBI Case 1: SDN1, SDN2) or a GMO (PBI Case 2: SDN3), depending on the use of a repair template and the nature of the insert. 

The decision tree followed a “20-bp rule”—this means that if an inserted sequence is 20-bp or more of a foreign DNA sequence, it is regarded as a GMO. However, an insertion of 19 bp or less of a foreign sequence is not regarded as a novel combination of genetic materials, hence the product is not subject to GM regulation. Additionally, GEd techniques that involve a repair template that inserts a 19-bp sequence and below will be considered as SDN-2, while those with an insertion of 20-bp or more will be considered as SDN-3. The SDN-2 edits will automatically be exempted from GMO regulation since the short insertion is not regarded as a novel combination of genetic material. In addition, other products of NBTs/PBIs that possess remnant border sequences from a transformation vector will be assessed based on the length of such sequences. The 20-bp rule described here was motivated by the report of the Joint Research Center of the European Commission (2011), and by the 20-bp guide sequence (crRNA) requirement in CRISPR-Cas9 systems that allow *Streptococcus pyogenes* Cas9 in nature to recognize and digest invading viral DNA. 

After the issuance of the NCBP resolution, the Department of Agriculture commissioned a Technical Advisory Group on Modern Biotechnology and related innovations for agriculture and fisheries (April, 2021). This resulted in a draft memorandum entitled “Rules and Procedure to Evaluate and Determine When Products of Plant Breeding Innovations (PBIs) or New Plant Breeding Techniques (NBTs) Are Covered under the DOST-DA-DENR-DOH-DILG Joint Department Circular No. 1, series of 2016 (JDC1) based on the NCBP Resolution no. 1, series of 2021”, which was finally published as Memorandum Circular No. 8 (MC8) in May 2022, ending a 4-year journey since the creation of the Study Group under DA-BPO. 

As per MC8, there will be a Technical Consultation for Evaluation and Determination (TCED), which is a technical evaluation of a PBI product to determine whether or not the final product is or is not a GMO. The process will be carried out by members of the Bureau of Plant Industry (BPI) Biotechnology Core Team (BCT-PBI) and assigned external expert(s). When a product has been officially determined to be a non-GMO, the team will issue a “*Certificate of Non-Coverage from the JDC1,* s2021” to the product developer. The certificate essentially recognizes the PBI product as being a conventional product, indicating that there is no need to obtain a Commercial Propagation Permit under JDC1, s2021 to register it as a variety or to be commercialized. A locally developed GEd crop, which is expected imminently to undergo TCED evaluation is bacterial leaf blight-resistant rice being co-developed by the International Rice Research Institute (IRRI) and the Philippine Rice Research Institute (PhilRice). 

### 2.9. The Regulatory Status of GEd Crops and Produce in Malaysia

The *Malaysian Biosafety Act* 2007 and the *Biosafety (Approval and Notifications) Regulations* 2010 served as the national guidelines for regulating LMOs in Malaysia. The former fulfils Malaysia’s obligation as a party to the *Cartagena Protocol* on Biosafety. Before the Act was tabled and the regulations came into force, the lead government agency, the *National Resources and Environmental Ministry* (NRE), consulted various stakeholders including the Malaysian Biotechnology Corporation, the Malaysian Biotechnology Information Center, the Malaysian Manufacturers Association, Consumer’s Association, and other Non-Governmental Organizations (NGOs). 

The *Biosafety Act 2007* describes the framework of the LMO regulation system; its overall objective is to protect humans, plants, animal health, and the environment by regulating the release, importation, exportation, and contained use of LMOs. Among others, the Act prohibits LMO activities to be conducted unless proposers have received prior approval or provided notification and the possible risks have been assessed. On the other hand, the Biosafety (Approval and Notification) Regulations 2010 contain additional information on the Act including how an application for approval for release or import activity must be made and matters relating to the certification of approval.

In Malaysia, a LMO is defined as a living organism that possesses a novel combination of genetic material through the use of modern biotechnology (defined as the application of in vitro nucleic acid techniques including recombinant DNA or fusion of cells beyond a taxonomic family) excluding traditional breeding and selection techniques. Currently, only a few activities are exempt such as pharmaceutical products of LMOs with relevant international treaties, organizations, or under written laws such as genetically engineered vaccines. However, the products cannot be released intentionally into the environment. Field trials involving LMOs growing in an open environment are considered as a ‘release activity’, and approval must be obtained before the trials start. 

If the product of an LMO is to be commercialized, it needs to be clearly identified and labelled. In 2010, the *Food (Amendment) Regulations* 2010 was passed, requiring all GM food and ingredients to be labelled before release into the market. However, a USDA Foreign Agricultural Service Report in 2018 noted that while Malaysia does not have any large-scale domestic production of GM crops, the country has expanded the number of GM products for commercial use, with more than 30 products being approved for import and market release (https://bch.cbd.int/en/countries/MY; Biosafety Clearing House, Malaysia, Conventional on Biological Diversity; accessed 30 July 2022). In addition, although mandatory labelling guidelines have been established, they have yet to be enforced (https://www.biosafety.gov.my/wp-content/uploads/2021/08/Garis-Panduan-Pelabelan-Makanan-dan-Bahan-Makanan-Melalui-Bioteknologi-Moden.pdf). According to the report, GEd plant products represent a very small fraction (if any) of the imported biotech produce and there is no commercialized production of GEd crops to date. Past projects include a confined field trial of GEd-papaya with delayed ripening. Interestingly, Malaysia is a significant importer of GM products for livestock feed such as soybean, demonstrating that the agricultural community supports the expanded use of GM products. A United Nation’s survey in Malaysia indicated that while industry and academia had a reasonable knowledge of the biosafety laws and regulatory framework, domestic consumer awareness was low. 

Recognizing the increasing importance of GEd products, the National Biosafety Board (NBB) has been actively engaging stakeholders to improve current regulations and to encourage research on GEd crops and produce in Malaysia. In reviewing the current regulations, the NBB has divided the regulatory scope into: from laboratory (notification) to market (approval) as well as into the three types of modifications, namely, SDN-1, SDN-2, and SDN-3. The division of regulations for each category is provided in Table 5.

#### Islamic Perspective for GEd Regulation of GE Produce in Malaysia

Malaysia has a diverse population: several groups have advocated the use of GEd technologies from an Islamic Fiqhi perspective. They argue that the country should have a comprehensive Islamic framework since Islam is the official religion and there is a Muslim majority in Malaysia. While this perspective focuses mainly on GEd for medical treatment, it would not be surprising if religious approval or certification is sought by consumers before consuming GEd products, for example, whether the crops are halal. Although this has not been discussed by the NBB, as GEd products become more established in Malaysia, this aspect may become more important as it reflects Malaysia’s culture and religion.

### 2.10. The Regulatory Status of GEd Produce in Indonesia

Indonesia, through the *National Biosafety Committee for Genetically Modified Organisms*, recognizes GEd as an important breakthrough that will play a significant role in its economy. As a megadiverse country with abundant bioresources, it is expected that the agriculture sectors (farming, forestry and fisheries) will benefit from the application of GEd technology as leading economic drivers, employing 29% of the total workforce [23]. 

In Indonesia, the earliest published reports on R&D on GEd in agriculture were in 2020 on the rice GA20ox-2 gene (cvs Kitaake and Mentong) encoding a gibberellin biosynthetic enzyme [24,25,26] and the OsCKX2 gene in the rice cv Mentik Wangi [27]. Several research institutes and universities are working on the GEd of different crops. For example, to improve the productivity of rice and soybean and cassava storage tolerance at the Research Center for Biotechnology-Indonesian Institute of Sciences, and improve the resistance to bacterial leaf blight in rice, resistance to geminivirus in chili pepper, increasing artemisinin content in artemisia, and resistance to citrus vein phloem degeneration (CVPD) in oranges at the Indonesian Center for Agricultural Biotechnology and Genetic Resources Research and Development (ICABIOGRAD) of the Ministry of Agriculture, and orchid flowering quality and disease resistance at the Universitas Gadjah Mada. 

The *Indonesian Institute of Sciences* (LIPI) and ICABIOGRAD have been integrated into the newly established *Indonesian National Agency for Research and Innovation* (BRIN). The funding for GE R&D is obtained mainly from the government of Indonesia: some activities are supported by Japanese agencies (JICA/JST and JSPS). In Indonesian GEd crops, the produce and research have not been specifically regulated. The Ministry of Agriculture and the then LIPI in collaboration with the *Biosafety Commission for Genetically Modified Product* (KKH-PRG) organized focused group discussions (FGD) attended by regulators and scientists from Ministries, Government agencies, and universities to try to reach a consensus on Indonesia’s stance on GEd issues and regulations. The first FGD, conducted by ICABIOGRAD in February 2019, discussed the progress of GEd research in Indonesia and worldwide, and the state of regulation in Indonesia. The FGD noted that GEd was being undertaken at ICABIOGRAD, the Research Center for Biotechnology, LIPI, the Indonesian Research Institute for Biotechnology and Bioindustry (PPBI), and the Universitas Gadjah Mada (UGM) on diverse commodities (rice, orange, palm oil, artemisia, and orchids), and for different purposes such as increasing productivity and stress tolerance. The FGD acknowledged that, globally, GEd products were reaching the stage of commercial release. The FGD also acknowledged that GEd would play an increasingly important role, and because no regulations existed, there was the need to develop appropriate regulations to govern the conduct of GEd research, the process for the release of GEd products, and to resolve any potential confusion on the definitions of the GM and GEd processes and products. 

The second FGD on GEd (20 February 2019), organized by the Directorate for Conservation of Biodiversity (Ministry of Environment and Forestry) and the *Indonesian Commission on Biosafety of Genetic Engineering Product* concluded that GEd products differed from GM products, and that there needed to be a clear definition of GEd to differentiate it from GM products. The FGD noted that since GM may be involved in the early development of some GEd products, some mechanisms of GM product regulation were in place, and could be applied for early assessment of the GEd products to determine whether the final products fall under GM product regulations. 

**Figure 11:**
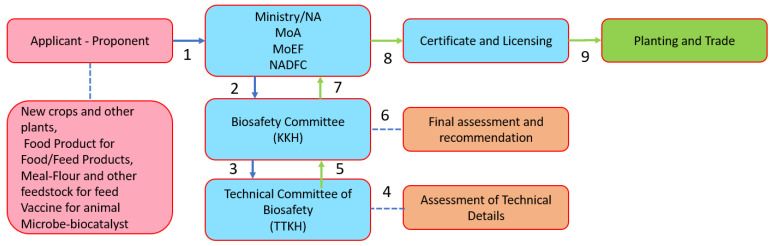
The procedures of the Biosafety Certification of new agricultural products in Indonesia. (The process follows the numbers as indicated, starting at the Ministerial level, proposal reviews by the Biosafety Committee and its Technical Committee, which assesses technical details, make a final assessment and recommendation for approval at the Ministry level, for issuing a certificate for licensing for planting or trade).

The definition of GM/genetic engineering in Indonesia is stated in Government Regulation No. 21/2005 on the Biosafety of Genetically Modified Products as “any living organism, part of it and/or its processed products with new genetic make-ups resulting from the application of modern biotechnology”. The definitions of LMOs and “modern biotechnology” were in line with the definitions under the *Cartagena Protocol* in which LMOs mean any living organism that possesses a novel combination of genetic material obtained through the use of modern biotechnology, and which involves the application of (a) in vitro nucleic acid techniques including recombinant deoxyribonucleic acid (DNA) and direct injection of nucleic acid into cells or organelles, or (b) the fusion of cells beyond their taxonomic family, which overcome natural physiological reproductive or recombination barriers, and that are not techniques used in traditional breeding and selection. In Indonesia, the decisions on GM products fall under the responsibility of the KKH-PRG, assisted by the *Technical Team for Biosafety for Genetically Modified Products* (TTKH-PRG). The TTKH-PRG consisted of TTKH-PRG for food (coordinated by the *Agency for Drug and Food Controls* (BPOM), TTKH-PRG for feed (coordinated by the Ministry of Agriculture), and TTKH-PRG for environmental safety (coordinated by the Ministry of Environment and Forestry). The flow of assessment is shown in Figure 11.

The definition of GMOs/LMOs was discussed at the third FGD (at the Indonesian Institute of Sciences, LIPI) in January 2020. This FGD, attended by members of the Indonesian Biosafety Committee, the Technical Team for Biosafety, the heads of biotechnology-based associations such as the Indonesia Biotechnology Consortium, Indonesia Biotechnology Society, and Indonesian Association for Agriculture Biotechnology, the Deputy for Life Sciences of LIPI, the Deputy under the Indonesian Agency for Drug and Food Control, the Director of the Research Center for Biotechnology-LIPI, the Head of BBBiogen-MoA, the Deputy Assistant under the Coordinating Ministry of Economy, and scientists from universities and research institutions, discussed decisions made at the previous FGD and shared experiences of regulators and scientists from three other countries. The FGD also discussed the need to reassess the definition of GMOs in Government Regulation No. 21/2005 to accommodate GEd products, which although developed mostly using modern biotechnology, could result in products that can be categorized as non-GMO. In other words, the FGD acknowledged that the process of producing a product via modern biotechnology could result in a non-GM product, depending on its genetic make-up (Table 5). The modern biotechnology products categorized as SDN-1 were determined to be non-GM because of their similarity with commonly occurring natural mutations. The policy on SDN-2 and SDN-3 products is still under discussion. However, the view was expressed that products of SDN-2 and SDN-3 could also be considered as non-GM when they fall into particular categories. For example, SDN-2 could be considered as non-GM as long as the template is from the same gene pool and no novel protein was produced. SDN-3, which is a knock-in mutation, could be considered as cisgenic, in which case the donor gene or DNA fragments come from the same gene-pool (Table 5). The decision on whether cisgenics are considered as non-transgenic, thus non-GM, are still under discussion.

**Figure 12:**
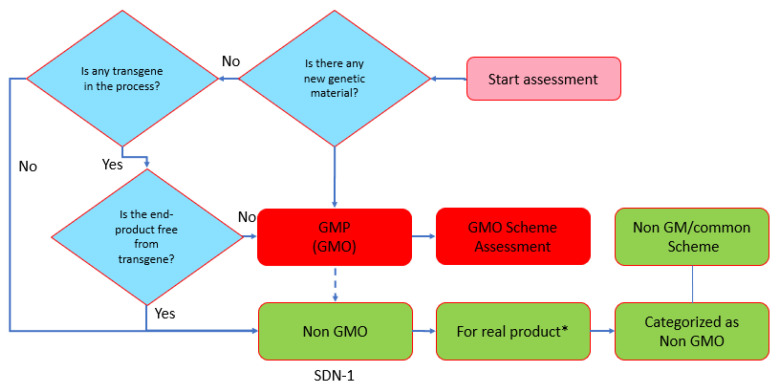
The mechanism of assessment of GEd products in Indonesia (* note for hypothetical products, there needs to be supporting data of molecular analysis and phenotype).

The FGD recognized the importance of GEd products to the Indonesian economy and recommended that a harmonization of the regulations/technical guidelines between GM and GEd products was needed, even before regulations on GEd were established, so that GEd products can be filed for release using the existing guidelines. This would require stating that the product is GEd and a non-GM, backed with scientific evidence. The FGD also recommended that a task force for the assessment, consisting of TTKH-PRG members and renowned scientists in the field, should be appointed to provide scientific recommendations to KKH-PRG. The scheme for handling the release of GEd products in Indonesia, also discussed during the third FGD, followed the available scheme for GM products, which requires the proponent to file a request for product assessment to the Agency for Drug and Food Control for food, to the Ministry of Agriculture for feed, and/or to the Ministry of Environment and Forestry for environmental safety. The responsible Ministry or Agency will request the KKH-PRG assess the status of the products for their GM status or if applicable, assess provided scientific data to support the non-GM status of the product. A recommendation would then be issued for the product to be released as for non-GM products. Since the third FGD, KKH-PRG has received one application for assessment of a GEd product, based on the SDN-1 approach. However, no decisions on GEd products have been made thus far. A flow diagram for the assessment process is provided in Figure 12.

### 2.11. The Regulatory Status of GEd Produce in Taiwan

In Taiwan, the regulatory policy on GE products is being reviewed by an *ad hoc* expert committee convened by the Taiwan Food and Drug Administration. Currently, mandatory pre-consultation and notification is required for GE foods and basic/safety information (i.e., evidence of no foreign DNA, or adverse effects) and reference materials need to be submitted for review, based on the draft policy disclosed in 2021. The cultivation of GEd products in Taiwan will be controlled under the *Plant Variety and Plant Seed Act* administered by the Council of Agriculture. The definition of whether GEd products are captured under this regulation is also not clear, and any new policy will need to clarify whether there will be exemptions for any GEd products. 

### 2.12. The Regulatory Status of GEd Produce in South Korea

The South Korean regulatory authorities have taken the view that GEd products are currently captured under the existing Transboundary Movement, etc. of Living Modified Organisms Act (commonly referred to as the *LMO Act*), which governs GM products. The *Act* is currently under review by five regulatory agencies. To exempt any GEd product from regulation, it would be necessary to introduce new amending regulations.

In May 2021, the Ministry of Trade, Industry, and Energy (MOTIE) released a draft revision of the *LMO Act*. The final decision for the exemption of GEd products from the regulations is presented after a pre-review by a Preliminary Review Committee, consisting of government regulatory agencies. Under Article 7–3 of the draft *Preliminary Review of New Living Modified Organism*, a new LMO may be exempt if:

- It has been made without introduction of foreign genes;
- Foreign genes are not retained in the final product; or
- The scientific evidence presented is credible enough to support that the final LMO developed by modern bioengineering technology, other than those set forth in Sub-paragraphs 1 and 2, can also be made by traditional breeding or natural mutation.

However, under the new Article 7–4 (Information collection for safety management of organisms), additional information may be requested for a preliminary review including the detection method and reference material.

The *Preliminary Review Committee* will consist of representatives of seven different Ministries. This new constitution of the committee could make assessments and the review process difficult (the current review system for GM products involves only five agencies). In addition, the introduction of a new category of ‘new genetically modified organisms’ appears unhelpful in the growing GEd field. Consultations on the draft are ongoing.

## 3. The Commercialization of GE Crops in Asia and Australia

The adoption of GM crops, which still hold much promise for addressing production challenges, has been severely hampered by strong opposition from anti-GM groups, leading to a lack of public/consumer acceptance. This has meant that in Asia as well as elsewhere globally, the cultivation and commercialization of GM crops has been confined to a few crop types, mainly cotton, corn, and soybean, and a limited number of countries. In the Asia-Pacific region, there is only meaningful cultivation in China (GM cotton), India (GM cotton), Pakistan (GM cotton), Vietnam (GM corn), the Philippines (GM corn), and Australia (GM cotton and canola). Apart from Australia, these countries import large amounts of grain from GM sources, mainly for feed and oil, and are important global markets for GM crops produced in both North and South America. The global grain trade is a directional flow of GM exports from the Americas to the leading markets of China, Japan, and Korea. However, the markets of some South-East Asian countries have become increasingly significant and are now important grain destinations for GM soybean and corn.

When trading GM commodity crops, regulatory approvals are a necessity and are already in place for currently traded products in the importing countries. Before commercializing a GM commodity crop, technology provider companies analyze the trade patterns and identify key export countries to make sure that the approvals are in place before launching in the country of cultivation. A recent study determined that the costs of discovery, development, and authorization of a new GM trait is in the vicinity of USD 115 million and takes about 16.5 years [1]. This expense, both in terms of time and money, practically rules out the ability for academic institutions and small- to medium-sized companies to commercialize GM commodity crops.

In contrast, crop traits developed using GEd, if not subject to the same regulatory constraints as GM crops, have the potential to offer an alternative solution to the production problems faced by farmers in the Asia-Pacific region. Because of the high regulatory cost to launch GM crops, commercialized events have been mainly limited to broadacre commodity crops where the seed market is big enough to recoup the up-front investments. If the regulatory costs for GEd crops are much lower than for GM crops, investment costs will be lower, making the investment in minor crops such as vegetables a real possibility. This would also increase the opportunity for the commercialization of GEd crops developed by local research institutions in Asia-Pacific countries, and may be brought to market by small- or medium-sized companies for regional production. This is already happening in the examples of the GABA tomatoes from Sanatech Seed and the GEd Madai sea bream from the Regional Fish Company in Japan. There is major R&D on GEd crops in China and India, although research in South East Asia on GE crops is still at an early stage. However, there is recognition of the potential of the technology, with governmental and semi-governmental research organizations having active programs in crops of local importance such as palm oil, driven by the hope that the regulation of GEd crops will not mirror that of GM crops.

In short, how GEd products are regulated in the Asia-Pacific will determine how much of the current and future research pipelines can become a commercial reality. As described above, there is mixed news regarding the regulation of GEd crops in SE Asia and Australasian countries. While uncertainty still remains for many countries, it appears that most governments have recognized that GEd technology in agriculture can play a big part in sustainability and production goals in the face of climate change, and there have been some developments in ASEAN countries (Singapore, Malaysia, Philippines, Indonesia, Myanmar, Vietnam, Laos, Cambodia, Thailand, Brunei) that support the view that GEd products that are equivalent to what can be developed through more conventional means should not be additionally regulated. The final position taken by a large block such as the EU may also influence the final policy decisions in ASEAN countries.

### 3.1. An Overview of GEd Technology Regulations in Asia and Australasia

**Figure 13:**
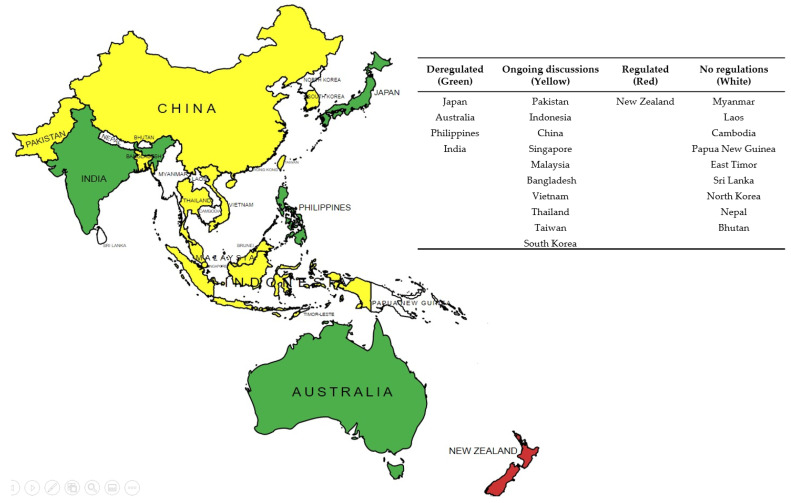
The regulatory status for GEd crops in countries in the Asia-Pacific region. It is based on the deregulation of SDN-1 crops (green), with some countries also deregulating SDN-1 and SDN-2 products, as described in the text. Countries with ongoing discussions (yellow) and regulated as GMOs (red). Note that regulation of GEd crops in China is under discussion, but does not use SDN terminology: at present GEd is still under GMO product safety management measures, but with less onerous requirements in the pathway to commercial approval.

Figure 12 provides and overview of the regulatory status of countries in Asia and Australasia. In summary, there is not yet a harmonized or uniform regulatory system for GE products in these countries to support the smooth international trading of agricultural products. The regulatory status of SDN-1 crops appears settled for a few countries, namely, the Philippines, Japan and Australia, which exempt some GEd products, and New Zealand (regulates GEd products as GM) (Figure 13). Some countries such as Singapore and Indonesia indicate that they will exempt some GEd products from regulations, while Thailand may opt for reduced regulatory requirements. 

The good news for advancing GEd technology is that the overall trend is clearly toward either exemption for some types of GEd products or toward fewer regulatory requirements compared to the GM products.

### 3.2. Lessons on GE Regulations, Trade and Harmonization from Other Parts of the World

For countries in Asia and Australasia keen to apply the GEd technology to improve crops, the goal is not only to provide food security for the nation, but also increases the quality and tonnage of their major export crops. To achieve that, the regulatory climate of GEd crops should be similar to those of the importing countries. Many countries have developed systems that make the current trading of GM crops possible, and as Asian and Australasian countries want to trade in the international market in the future when GEd crops become acceptable commodities, it will be imperative that lessons from successful countries are learnt and decisions on the deregulation of GEd crops are taken with a view to brevity and clarity. Examples from nations around the world are discussed below.

#### 3.2.1. Using Established and Trusted Frameworks

The two major economies in North America, the United States of America (USA) and Canada, have moved swiftly to support the development, deregulation, and commercialization of GEd crops, mainly by employing the framework established successfully and implemented to regulate GM crops. As the largest grower of GM crops [28], the USA was the first country to approve a GEd product for commercial sale. This was Calyxt’s GEd soybean with no trans-fats and lower saturated fat produced using TALENs [29]. The enthusiastic uptake of GEd technology in the USA perhaps reflects that even the introduction of GM technology in the 1990s did not trigger the need for new regulations, but instead relied on applying the existing regulatory frameworks to oversee these new crops. The adoption of such a strategy by Asian and Australasian countries could remove barriers and red tape, which has slowed decision-making on deregulation, even of SDN-1 crops, viewed as the least complicated of the outcomes of GEd technology. 

In the USA, up to three different agencies can be involved in the regulatory oversight, depending on the final product and how the plant was produced. These are the U.S. Department of Agriculture’s ‘*Animal and Plant Health Inspection Service*’ (USDA-APHIS), responsible for protecting agriculture from pest and diseases, the *Environmental Protection Agency* (EPA), which regulates pesticides and therefore regulates biotech crops that have pesticide properties (e.g., insect resistant crops, where they are considered plant incorporated protectants (PIPs)), and the Food and Drug Administration (FDA), which oversees food safety. The FDA consultation process is voluntary, and the result of this consultation is an acknowledgement from the agency that there are “no further questions” concerning human or animal food derived from the GM plant based on the information provided by the applicant. 

In 2020, the USDA-APHIS finalized its SECURE (Sustainable, Ecological, Consistent, Uniform, Responsible, Efficient) rule, which exempts certain GEd plants that otherwise could have been developed through conventional breeding. This focuses on regulating the properties of GEd plants, rather than the process used to create them. APHIS states that the exemptions are intended to bring the regulation of potential GEd plants more in line with the guidelines for conventionally bred crops. Therefore, GEd crops that do not contain foreign DNA are not regulated as GMOs if they pose no risk to other plants, and show no food safety attributes different from those of traditionally bred crops. It becomes the responsibility of the developer to assure that products to enter the market are safe for use and consumption (as in the case for conventional crops). The FDA and EPA are yet to announce whether their existing policies and regulations, related to GMOs, would be used to regulate GEd crops and food. Further guidelines for requesting a *Regulatory Status Review* (RSR) can be found on the APHIS-USDA website.

Like the USA, Canada has a well-established product-orientated approach to policy and regulatory oversight and regulates all plants with novel traits (PNTs), regardless of the technology used to create them. Health Canada considers that GE technologies do not present any unique or specifically identifiable food safety concerns compared to other technologies of plant development. Therefore, GEd plant products should be regulated like all other products of plant breeding within the Novel Food Regulations (i.e., by the novel traits and how these traits impact food safety). The health, product, and trait-based strategies used for the deregulation of GEd crops in the USA and Canada, if adopted, will help smaller economies in Asia and Australasia focus their research funds and time on traits important for their economies. GEd traits of such significance are likely to be accepted by consumers, possibly forcing governments to deregulate their use. 

#### 3.2.2. Modified and Progressive Assessment and Deregulation

The evidence that positive changes in GEd regulations can be achieved is exemplified by the UK. The 2001/2018 EU Court of Justice directive ruled that a GEd product was to be regulated as a GMO within the EU (which then included the United Kingdom, UK) and would not fall under the mutagenesis exemption of the directive as for older established mutagenesis technologies with a safe history of use. The UK formally left the EU in January 2020, and this gave the UK scope to move from the restrictive GMO EU Directive and set its own regulatory path. Within the UK, England, Scotland, Northern Ireland, and Wales have national laws that control the release of GMOs into the environment. In England, the Department of Environment, Food, and Rural Affairs (Defra) is responsible for the environmental release of GM plants, with all applications submitted to Defra also being passed on to the statutory Advisory Committee on Releases to the Environment (ACRE) appointed under Section 124 of the *UK Environmental Protection Act* 1990 (EPA) to provide advice to the Government regarding the release and marketing of GMOs. The committee works within the legislative framework set out by ‘Part VI of the EPA’, and in England, the *GMO Deliberate Release Regulations* 2002 *Act*, which together previously implemented EU Directive 2001/18/EC. The principal role of ACRE is to consider each application on a case-by-case basis and evaluate the risks to human health and the environment.

Defra launched a consultation exercise in early 2021, resulting in an announcement by the UK Government on Genetic Technologies (published 29 September 2021) for a two-step reform. The first step removed the regulatory burden for research groups by enabling the field trials of GEd crops (free from transgenes) to go ahead without being subject to existing GMO rules, under a new on-line ‘notification’ system to Defra. The second step was to bring forward primary legislation to amend the regulatory definitions of a GMO and to exclude organisms that have genetic changes that could have been achieved through traditional breeding or that could occur naturally [30]. These crops would then be regulated in line with conventional crops, with ‘novel food’ oversight [31] where appropriate. This would enable much easier trade relationships with counties that have adopted similar regulations. However, the impact on trade with the EU is still a hurdle, as the UK regulations differ from those in the EU. It should also be noted that currently, the changes to legislation apply only to England: how that may impact trade with Wales and Scotland is not yet clear. 

Another example of recent progress in GEd policies and regulations is that two countries in Africa have now deregulated GEd crops: Nigeria and Kenya. As the first country in Africa, Nigeria has authorized guidelines on GEd in December 2020 through its National Biosafety Management Agency. Decisions will be made on a case-by-case basis: if edited lines do not contain a new combination of genetic material, they can be classified as conventional varieties or products. In February 2022, Kenya’s National Biosafety Authority published guidelines that provide the framework for exemptions of GEd organisms and products from the Biosafety Act, enabling a case-by-case approval that would treat them as conventional varieties or breeds [32]. Malawi, Ethiopia, and Ghana are also currently developing their policies, while South Africa is currently in an on-going appeals process after a decision to consider all GEd plants as GMOs.

Similarly, for Norway, it has been proposed that GEd crops that do not contain DNA from another species be regulated as conventional plants, but would still require notification. In Israel, GEd crops that do not contain DNA from another species are also regulated as conventional plants. The status of GEd plants has yet to be defined in the Russian Federation [33]. A similar approach is adopted by most South and Central American countries where GEd crops are regulated as conventional plants unless they contain foreign DNA: in some countries, notification to the authorities is required to approve this exemption [34,35].

#### 3.2.3. Avoiding the “Protection without Clarity Regulation”

It is to be hoped that the lessons from the European Union’s (EU) approach to regulating GM and now GEd crops will be avoided by countries in the Asia-Pacific region. This relates to the isolated approach adopted by the EU to regulate GM/GEd crops, where crucial definitions leave room for diverse interpretations, while at the same time, scientists in the community and some member countries are not on board with the regulations. 

Before 2018, several Member States (e.g., Sweden and the UK, then being part of the EU) had interpreted the mutagenesis exemption to the 2001/18 EU GMO directive to include precision mutagenesis applications such as GEd crops, that had been edited in a way that would result in a product indistinguishable from one obtained through traditional mutagenesis techniques (i.e., chemical or radiation induced mutagenesis). 

In 2016, nine NGOs filed a case to the French Courts, (later referred to as the CJEU), to challenge the interpretation of the earlier exemption on GEd (EU GMO directive [36]) to allow ‘GM through the backdoor’. Following the 2001/18 EU GMO Directive, over 117 research facilities signed a position paper urging the European Policy Makers to act to safeguard Europe’s competitiveness on these new technologies [37]. For many, the ruling fell short of delivering clarity on the regulatory status of GEd, and how such crops would be monitored [38]. 

The EU Council later requested that the EU Commission look in more detail at the impact of the CJEU ruling. The results, published in April 2021, concluded that the current GMO legislation was ‘not fit-for-purpose’ for some NGTs and their products, and that it needed to be adapted to keep in line with scientific and technological progress [39]. The lack of clarity surrounding the future regulatory climate for GEd crops has resulted in several EU-based companies focusing on developing GEd crops for non-EU markets [40]. How the EU will be able to implement the regulation and traceability of such crops is also not clear. This debate will continue for the EU, and at the time of writing, a public consultation was open to gain feedback on a proposed new legal framework for plants obtained by targeted mutagenesis and cisgenesis, and for food and feed products. However, any new legislation for review is not expected before Q2 2023. The EU situation is a salutary lesson that Asia-Pacific countries would do well not to follow the EU example on regulating GEd crops.

### 3.3. Issues Relating to the Commercialization of GEd Plants

#### 3.3.1. Differing Definitions of SDN-2 in Different Jurisdictions

From the information provided above, it is evident that although national definitions of SDN-1 and SDN-3 products of GEd are clear, those for SDN-2 products are not. An overview of SDN-2 definitions in Asia and Australia is provided below, together with comparisons from the EU and the UK (Table 6).

#### 3.3.2. Proving the Absence of External DNA or New Allergenic Peptides in SDN-1 Plants

One issue relates to the method used to generate SDN-1 plants. Often, the editing machinery (Cas protein and guide RNAs) are introduced as a T-DNA. Once a successful edit is identified, the plant is selfed and selected for null segregants (i.e., plants containing the edit but no longer containing the T-DNA). However, the introduction of undesired DNA sequences from the plasmid backbone might still occur. The question follows, how much effort needs to be made to determine whether there is introduced DNA or not? SDN-1 plants with no additional T-DNA may legally be grown commercially, but if introduced DNA is subsequently found, the plant may become a GMO, and growing that plant without a license may then become illegal. It remains to be determined how much effort should be put into demonstrating a negative, that is, that there is no added DNA. At the very least, for the commercialization of SDN-1 edited plants, the plants would be sequenced at the site of editing to show that no vector DNA is present, and quantitative PCR used to identify the absence of any backbone DNA. In addition, the sequences surrounding the edit site should be checked using appropriate databases to show that no new small ORF formed (e.g., >30 amino acids) and that no new allergenic peptides resulted. These considerations emphasize the need to keep good records and the importance of the stewardship of relevant data.

#### 3.3.3. Off-Target Edits

It is important to view any off-target edits of GEd in context. Spontaneous mutations occur naturally at rates of ~10^−8^ to 10^−9^ per site per generation, and pan-genome sequencing has revealed that many sequence differences exist within a species [41]. There are also more than 3200 varieties generated by induced mutagenesis, not counting the variation caused by somaclonal variation [42]. Off-target GEd may occur at sites similar to the target site, if present. Off-target edits can be minimized by checking the genomic sequence of the plant if available and choosing alternative guide RNA sequences. Recently developed CRISPR editing systems are also more specific, and various studies have indicated that the proper design of gRNAs leads to undetectable levels of off-target edits [43]. Studies on rice [44], cotton [45], and maize [43,46] attributed nearly all of the variation in re-sequenced GEd plants to tissue culture-induced somaclonal variation. This is similar to findings for transgenic plants [47]. The history of the safe consumption of foods from plants is based on the fact that many mutations, regardless of origin, have no phenotypic effect. In conventional breeding, these neutral genetic changes cannot be removed from plant populations. Clearly, possible off-target edits in plants present much-reduced safety concerns compared to those that might arise in medical applications of GEd [41], and off-target edits that result in an undesirable phenotype will routinely be eliminated in subsequent crop breeding.

#### 3.3.4. Unintentional Low-Level Presence of Edited Seeds

An SDN-1 plant may be approved for growth in one country, but not in a country that imports such seeds. The low level presence (LLP) of GM or GEd products is more an issue of bulk grain transport, and of the asynchronous development of national regulations, but could disrupt trade. A group of countries have joined to form the Global Low-Level Presence Initiative (GLI: https://llp-gli.org/; accessed 2 July 2022). The GLI is composed of 15 importing and exporting countries committed to working collaboratively on approaches to minimizing unnecessary trade disruptions when addressing LLP, and includes some countries in SE Asia. These countries are Australia, Argentina, Brazil, Canada (member and Secretariat for the GLI), Colombia, Costa Rica, Indonesia, Mexico, Paraguay, Philippines, Russia, South Africa, United States, Uruguay, and Vietnam. Although formed to address the small amounts of commercialized GM grain, despite the best industry management practices, which might be present in shipments to countries where the use of the grain has not yet been authorized, it is also relevant to GEd grains for the same reasons.

#### 3.3.5. Patents and Licensing 

Any group considering the commercialization of a GEd crop or its products should seek professional and legal advice concerning the potential gene targets and the need for a license to use a particular GEd technology. It is beyond the scope of this review to provide such advice.

### 3.4. Science Diplomacy—A Pathway toward Regulatory Harmonization? 

Science diplomacy is a policy discussion in which the aim is to combine the values of science and diplomacy to help provide technical solutions to global challenges such as food security [48]. Biotechnology provides an example where science diplomacy can be used to facilitate effective science communication between stakeholders and promote impactful scientific outcomes in multilateral negotiations including the harmonization of regulatory triggers for plant-based GEd [49]. 

The multilateral treaty system and on-going negotiations at the United Nations and other regional organizations is likely to have a direct impact on the regulations related to the commercialization of biotechnology products. There is a real need to include current scientific knowledge on GEd products in the context of the Cartagena Protocol on Biosafety, which deals with the environmental release of biotech crops. Without clarity on the regulatory status of GE crops in the international treaty process, efforts to harmonize regulations will be more difficult and can lead to institutional drift [50]. The benefits of international GEd policy harmonization may be achieved more rapidly through negotiation simulations, science policy/diplomacy education [51], policy advocacy platforms [52], and grant structures [53]. 

Science diplomacy can therefore help increase the communication between policy/diplomacy communities at the level of product (regulatory triggers), process (international conventions), and education (science communication), thus reducing institutional drift [54]. Various organizations and networks such as the International Rice Research Institute (IRRI), International Maize and Wheat Improvement Center (CIMMYT), and the Consultative Group on International Agricultural Research (CGIAR) are in key positions to increase the impact of science advice in international treaties to promote harmonization. Science diplomacy can enable cross-sectoral treaty discussions and national capacities in developing regulatory guidelines related to agricultural biotechnology, its commercialization, and trade

## 4. Conclusions and Prospects for the Future

The current population in Asia and Australasia is about 4.75 billion people (about 60% of the world’s total population) and it is predicted to grow to 5.5 billion by 2050 (www.worldometers.info; accessed 2 July 2022). New GE technologies hold the promise to make a major contribution to increasing the world’s food supply for both farmers and consumers, and for human health and the environment. The frontiers of plant breeding are moving from transgenesis as a potentially dominant form of plant breeding, mainly because of the onerous and prohibitive regulations surrounding GMOs/LMOs. In contrast, developing regulations are treating GEd plants more as mutagenized crops, in a targeted rather than random way [55]. Since many thousands of commercialized crops derived from mutagenic treatments are available without regulation as GMOs, and which can also be sold as ‘organic’, the developing regulatory landscape for GEd crop products could mark the end of pointless battles over GMOs. There is now an opportunity to fundamentally alter the ‘risk–utility’ balance for GEd products [55].

However, it is clear that regulatory harmonization, or at least alignment, of GEd crops and produce is crucial, and this is particularly the case for Asia and Australasia. Without the harmonization/alignment of GE regulations, crop industries may well face the same trade issues that have limited the wider commercialization of GM crops. From the narrative provided above, it appears that increasingly more of the world’s nations are proceeding to a rational approach of regulating GEd crops, following the principle that like products should be regulated in the same way. Some have been influenced by EU policies, and some are swayed by NGOs and activists, rather than because they disagree with the science. Commonly accepted definitions of SDN-1, SDN-2, and SDN-3 would be a great help. With the aid of science diplomacy and meaningful international discussions, the harmonization or alignment of GEd regulations can be achieved, thus enabling the full benefits of GEd technologies to be realized.

## Figures and Tables

**Table 1:** The status of organisms with SDN-1 modifications in Australia by method of SDN application.

	SDN Protein Applied (with or without sgRNA)	SDN Expressed from a Transgene That Is Only Transiently Present in the Organism	SDN Expressed from Transgene Integrated in the Genome
Status of the initial organism modified by SDN-1	Not a GMO (Schedule 1 item 4)	GMO while transgene or its expressed products are present Not a GMO when transgene and expressed products have degraded (Schedule 1 items 4 + 10)	GMO
Status of offspring inheriting the SDN-1 modification	Not a GMO (Schedule 1 item 9(a))	Not a GMO (Schedule 1 item 9(b))	GMO if SDN transgene also inherited Not a GMO if no SDN transgene inherited (Schedule 1 item 9(b))

**Table 2:** A summary of the policies for handling food products derived from GEd in Japan.

Type of GE Outcome	Food Sanitation Law (MHLW)	Feed Safety Law (MAFF)	Cartagena Law (MOE/MAFF)
SDN-1: Deletion/insertion, no template	Non-GMO	Non-GMO	Non-GMO
SDN-2 Change with template	Non-GMO (1-few bp)	Non-GMO (1-few bp)	GMO/Non-GMO *
SDN-3 Addition of new genetic material	GMO	GMO	GMO/Non-GMO *

* Non-GMO: when the template DNA is from the same species (self-cloning/intragenic) or from sexually compatible species (‘natural’ occurrence). MHLW: *Ministry of Health, Labor and Welfare*, MOE: *Ministry of the Environment*, MAFF: *Ministry of Agriculture, Forestry and Fisheries.*

**Table 3:** Some ongoing R&D on GEd at Indian institutions.

Institutes	Crop	Trait
National Institute of Plant Genome Research, New Delhi	Indian Mustard	Glufosinate alkaloid reduction to tolerant level
	Rice	Disease resistance and herbicide tolerance
	Chickpea	Seed size and quality
	Rice/Maize-	Improvement of root architecture and stress/nutrient response/abiotic stress tolerance
Bose Institute	Tomato	Adjusting complex traits
Junagadh Agricultural University, Gujarat	Groundnut	High oleic acid and low linoleic acid
Indian Agricultural Research Institute, New Delhi	Rice	Yield, nitrogen use efficiency, water use efficiency, abiotic and biotic stress tolerance
International Center for Genetic Engineering and Biotechnology, New Delhi	Rice	Low phytate; nutrient use efficiency; herbicide tolerance
National Research Center on Plant Biotechnology	Indian Mustard	Seed meal quality
Tamil Nadu Agricultural University, Coimbatore	Rice	Disease resistance and nutritional quality
Institute of Life Sciences (ILS), Bhubaneswar	Bhimkol (*Musa* *balbisiana*)	Seedless
National Agri-Food Biotechnology, Mohali	Banana	Increase levels of beta carotene

**Table 4:** The current R&D on the GEd of crops in Pakistan.

Institute	Crop	Targeted Trait/s
National Institute for Biotechnology and Genetic Engineering (NIBGE)	Wheat	Yield improvement, disease resistance, nutritional enhancement
	Potato	Disease resistance and quality improvement
	Cotton	Quality improvement, biotic and abiotic stress tolerance
	Rice	Yield improvement, disease resistance, and herbicide tolerance
	Brassica	Edible oil quality improvement
Centre for Excellence in Molecular Biology (CEMB)	Tomato	Virus resistance
	Potato	Reduction in cold-induced sweetening, scab and blight resistance
	Cotton	Induction of male sterility and virus resistance
	Corn	Herbicide tolerance
Forman Christian College University (FCCU)	Wheat	Quality improvement
	Cotton	Heat/drought resistance
National Institute of Genomics and Advanced Biotechnology (NIGAB)	Potato	Reduction in potato browning
	Wheat	Yield improvement and root growth improvement
	Tomato	Enhancing shelf life
National Center for Genome Editing (NCGE), University of Agriculture	Wheat	Yield improvement and quality improvement
	Brassica	Edible oil quality improvement
	Cotton	Disease resistance

**Table 5:** The regulatory scope of the Biosafety Act in Malaysia for LMOs.

Notification—Part IV of Act	Approval—Part III of Act	
**Developing LMOs—From Bench to Market**		
**R&D**	**R&D**	**Commercialization**
Contained use of LMOs Import for contained use Export of LMOs	Field trials	Direct introduction to the environment. Commercialized planting. Placing in the market.
**Direct Commercial Use—No R&D**		
Export of LMOs Contained use for industrial production	Importation of LMOs/products for placing in the market or release	

**Table 6:** Differing definitions of SDN-2 in different jurisdictions.

Country	SDN-2 Definition	Comments
Australia	An organism modified by the repair of single-strand or double-strand breaks of genomic DNA induced by a site-directed nuclease, if a nucleic acid template was added to guide homology-directed repair.	SDN-2 products are still regulated as GMOs (this remains a fundamental point of disagreement between industry and the Regulator, which prefers a decision based on what changes this makes to the final product)
China	GEd crops in China do not fall into the categories of SDN-1, SDN-2	No clear equivalent definition available
Japan	Change with template of 1 to a few bps (the definition is ambiguous to leave room to implement a flexible policy)	SDN-2 is not regarded as GMO if the template DNA is from the same species or from a sexually compatible species.
Philippines	A gene-editing technique that inserts a maximum of 19-bp DNA sequence (foreign or non-foreign) from a repair template, producing a non-GMO.	The definition is different from most others
Thailand (draft definition)	A technique in which template DNA is used to modify a targeted DNA sequence to be an intended sequence modification as expected by homology-directed repair (HDR).	Legislation still under consideration. Maximum insert size proposed to be 10 kb.
India	In the recently issued ‘Guidelines for Safety Assessment of Genome Edited Plants’ 2022′, SDN-2 has been defined as site-directed mutagenesis using a DNA sequence template. Further elaborated in the ‘comments’ column	SDN-2 involves a template-guided repair of a targeted DNA break using an externally supplied template sequence. The donor carries one or several small mutations flanked by twosequences matching both ends of the DNA break, and is thus recognized as a repair template, allowing the introduction of the mutation(s) at the target site. The resultant mutant carries modified sequence, leading to altered expression profile of the gene and/or altered activity of the encoded protein/RNA. Thus, the edited version could be regarded as an allelic form comparable to those available in primary/secondary gene pool’.
Pakistan	No official definition	An agreed definition of SDN-2 is still under discussion in Pakistan.
Bangladesh	No official definition	GEd policies under discussion.
Indonesia	Targeting a specific location Non-GM SDN-2 products: (a) Use own gene pool, no novel protein (b) Use other gene pool as repair template, no novel protein	SND-2 Classified as GM if a novel protein is produced. SDN-3—cisgenics using own gene pool—under discussion whether non-GM or GM Use of another gene pool or production of a novel protein—regulated as a GMO.
Vietnam	No definition at present	
EU	In SDN-2 applications, specific point mutations, small deletions/additions are generated as a result of the introduction into the cell of a repair DNA template (donor DNA) homologous to the targeted area. By means of homologous recombination (HR), precise and small genetic modification can be achieved.	Regulated as a GMO.
UK	SDN-1 and 2 combined—changes that could have been produced by traditional breeding. Genetic Technology (Precision Breeding) Bill—Parliamentary Bills—UK Parliament under discussion.	Allowed to take SDN-1/2 to field trials without a GMO license—but need to notify Defra (competent authority). The aim of current legislation is to allow edits that could have been obtained by traditional breeding or in nature not to be viewed as GMOs.

## Data Availability

Not applicable.
